# Differential miRNA expression in *Rehmannia glutinosa *plants subjected to continuous cropping

**DOI:** 10.1186/1471-2229-11-53

**Published:** 2011-03-26

**Authors:** Yanhui Yang, Xinjian Chen, Junying Chen, Haixia Xu, Juan Li, Zhongyi Zhang

**Affiliations:** 1 College of Agronomy, Henan Agricultural University, 95 Wenhua Road, Zhengzhou, PR China

## Abstract

**Background:**

The productivity of the medicinally significant perennial herb *Rehmannia glutinosa *is severely affected after the first year of cropping. While there is some information available describing the physiological and environmental causes of this yield decline, there is as yet no data regarding the changes in gene expression which occur when the species is continuously cropped.

**Results:**

Using a massively parallel (Solexa) DNA sequencing platform, it was possible to identify and quantify the abundance of a large number of *R. glutinosa *miRNAs. We contrasted the miRNA content of first year crop plants with that of second year crop ones, and were able to show that of 89 conserved (belonging to 25 families) and six novel miRNAs (six families), 29 of the former and three of the latter were differentially expressed. The three novel miRNAs were predicted to target seven genes, and the 29 conserved ones 308 genes. The potential targets of 32 of these differentially expressed miRNAs involved in the main transcription regulation, plant development and signal transduction. A functional analysis of the differentially expressed miRNAs suggested that several of the proposed targets could be directly or indirectly responsible for the development of the tuberous root.

**Conclusion:**

We have compared differential miRNAs expression in the first year crop (FP) *R. glutinosa *plants and second year crop (SP) ones. The outcome identifies some potential leads for understanding the molecular basis of the processes underlying the difficulty of maintaining the productivity of continuously cropped *R. glutinosa*.

## Background

*Rehmannia glutinosa *L. is a perennial herbaceous species belonging to the *Scrophulariaceae *family. Its economic importance results from the medicinal activity present in extracts of its tuberous roots [1]. Because of a lack of known undesirable side effects and its relatively low price, the species is extensively used in traditional Chinese clinical practice. Its prime production region is the Huai area of central China, but the climatic and edaphic conditions in Jiaozuo (Henan province) are also conducive for the cultivation of a high quality product. After one season of production, however, disease build-up (and other factors) forces the land to be cultivated with other crops for a period of 15-20 years [2]. Even in the absence of disease pressure, attempts to continuously crop over several seasons have failed to overcome the major decline in productivity, as the tubers are increasingly replaced by fibrous roots, which are unable to develop into tubers [3,4]. Much of the past research aimed at identifying the causative factors for this continuous cropping yield decline has been focused on the physiological activity and autotoxicity of the root exudates [5-7]. However, the molecular basis of the species' sensitivity to its own exudate remains unknown.

miRNAs (short RNA molecules, on average ~21 nucleotides in length) underlie a number of biological phenomena in the animal, plant and virus kingdoms [8], largely at the level of post-transcriptional gene regulation [9-12]. As their sequences are so highly conserved across the eukaryotes, they are believed to represent an evolutionarily ancient component of gene regulation. They operate via their complementarity to a stretch of mRNA sequence, and affect the level of gene expression by targeting the mRNA molecule for degradation. The short stretch of sequence present in an miRNA means that many probably interact with a number of independent mRNAs. Commonly, the miRNA target sequence lies within a coding region, although there are examples of sites lying in either the 3' or 5' untranslated region [13-15]. The spectrum of functions now known to be miRNA-regulated is very diverse [16-20] and includes many aspects of plant growth and development [21-32].

Our hypothesis here was that miRNA activity may underlie some at least of the the problems associated with the continuous cropping of *R. glutinosa*. In order to gain a global picture of the miRNA content of *R. glutinosa*, we have therefore employed a high throughput parallel sequencing platform (Solexa sequencing) able to generate millions of short (18-30 nt) reads with a high level of accuracy. We have applied this technology to enable the comparative profiling of the miRNA content of plants in their first year of cropping (FP) with those in their second year (SP), with the intention of identifying miRNAs expressed differentially in FP and SP plants.

## Results and Discussion

### Sequencing and annotation of *R. glutinosa *miRNAs

**Figure 1:**
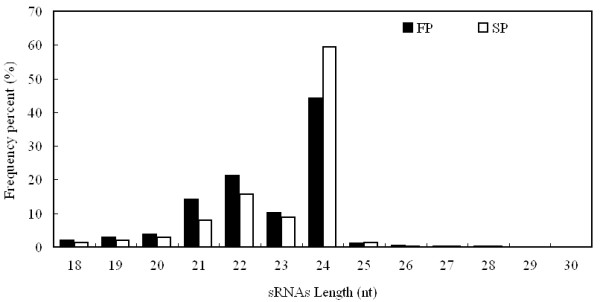
**Size distribution of *R. glutinosa *small RNAs**.

Solexa sequencing of the FP and SP miRNA libraries yielded, respectively, 17,619,697 and 18,028,647 unfiltered sequence reads. Of these 19.92% (unique 39.37%) were FP-specific, 23.82% (unique 48.17%) were SP-specific and 56.26% (unique 12.46%) were their common respectively. The average number of occurrences of the sequences common to both libraries was 10.5, while that of library-specific reads was not more than 1.2 (Table 1). After discarding the low quality reads, a total of 14,630,881 FP and 15,644,334 SP reads was retained. These sequences represented 6,748,998 and 7,894,661 unique clean reads in FP and SP, respectively. Their size distribution (Figure 1) showed that ~94% of the sequences in both libraries were of length 20-24 nt, with the modal length of 24 nt and the third peak at 21 nt, consistent with the observed length distribution of mature plant miRNAs [33,34].

**Table 1:** Small RNA sequences present in both FP and SP plants, and those specific to one or other plant type.

Class	Unique sequences	Percentage Percentage (%)	Total sequences	Percentage (%)	Mean frequency
**Total reads**	130,20,836	100.00%	30275211	100.00%	2.33
**FP and SP Common**	1,622,823	12.46%	17,033,751	56.26%	10.50
**FP specific**	6,271,838	48.17%	7,211,298	23.82%	1.15
**SP specific**	5,126,175	39.37%	6,030,162	19.92%	1.18

### Conserved miRNAs

**Figure 2:**
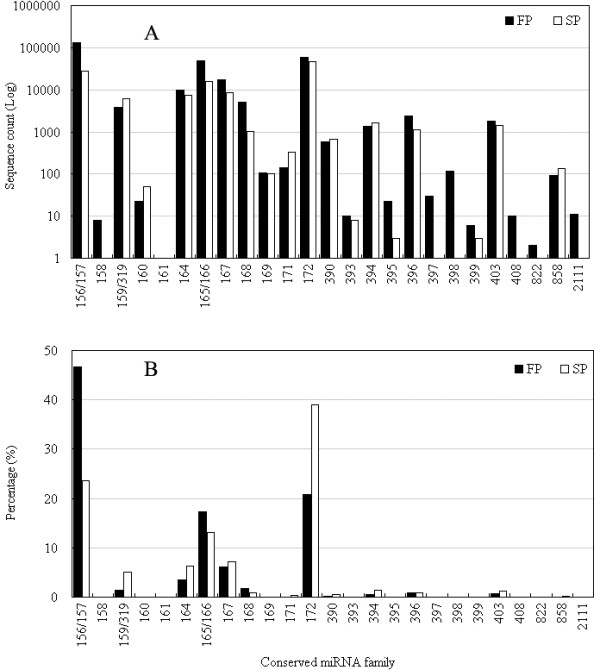
**The relative abundance of conserved miRNA sequences**. (A) The number of occurrences of a sequence. (B) The ratio between the number of sequences in FP (or SP) and the total number in the pooled library.

Sequences homologous to non-coding sequences (rRNA, tRNA, small nuclear RNA and small nucleolar RNA) were identified from a search of the GenBank and the Rfam9.1 databases. This resulted in the allocation of 0.93% of the FP and 0.63% of the SP unique miRNAs to this category. When the remaining sequences were queried against known miRNA sequences, the outcome was the identification of 282,063 (unique 300) and 118,011 (unique 251) hits, accounting for, respectively, 1.93% and 0.75% of the FP and SP libraries. A BLASTn search of the genic miRNAs resulted in the identification of 89 sequences, belonging to 25 families. The extent of their conservation across the plant kingdom was shown by an alignment with the whole genome sequences of *A. thaliana*, soybean, rice, black poplar and grape (Table S1 in Additonal file 1). The most abundant sequences were miR156/157, miR172 and miR165/166; the former accounted for ~47% of all conserved miRNAs in the FP library, while the most frequent single conserved sequence in the SP library was miR172 (~39%) (Figure 2). In both libraries, miR159, miR394 and miR403 were moderately abundant. The five miRNAs miR161, miR397, miR398, miR408 and miR822 were absent from the SP library. It appeared therefore that the miRNA population present in FP plants differed to some extent from that present in SP plants.

### Novel miRNAs

**Figure 3:**
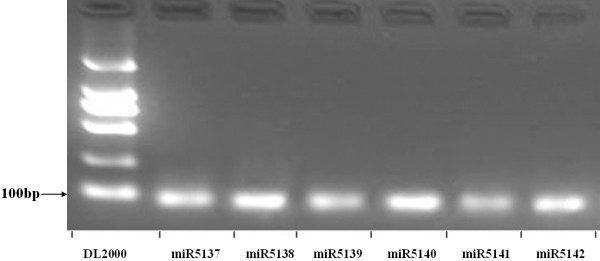
**RT-PCR products of novel miRNAs in *R. glutinosa***.

A distinguishing feature of miRNAs is the ability of their pre-miRNA sequences to adopt the canonical stem-loop hairpin structure. After removal of the conserved miRNAs, 13,724,517 FP (6,685,869 unique sequences) and 15,043,261 (7,845,823 unique sequences) SP sequences were aligned with the *A. thaliana* genome sequence, producing 7,341 (3,158 unique sequences) FP and 7,468 (3,269 unique sequences) SP sequences (Table 2) whose flanking region (in *A. thaliana*, at least) was amenable to secondary structure analysis. The application of a set of strict identification criteria for potential miRNA loci [35,36] resulted in the selection of 18 sequences (Additional file 2) across the two libraries which could be considered as likely novel miRNAs (Table S2 in Additional file 1). Except for miR5138, their frequency of occurrence was <40 (Table S2 in Additional file 1), reflecting an expression level considerably lower than that of the majority of the conserved miRNAs. When RT-PCR was applied to these 18 sequences, six were amplifiable from *R. glutinosa *cDNA template (Figure 3).

**Table 2:** Annotation of sRNAs sequences from SP and FP.

Category	Unique signatures		Total signatures		Mean frequency	
	
	FP	SP	FP	SP	FP	SP
**Non-protein-coding RNAs**	62,829 (0.93%)	48,587(0.63%)	624,301(4.26%)	483062 (3.08%)	9.94	9.94

rRNA	52,498 (0.78%)	39,983 (0.51%)	472,541 (3.23%)	346,969 (2.22%)	9	8.68

snRNA	1,461(0.02%)	1,304 (0.02%)	2,607 (0.02%)	2,158 (0.01%)	1.78	1.66

snoRNA	542 (0.01%)	513 (0.01%)	731 (0.00%)	709 (0.00%)	1.35	1.38

tRNA	8,328 (0.12%)	6,787(0.09%)	148,422 (1.01%)	133,226 (0.85%)	17.82	19.63

**Known miRNAs**	300 (0.00%)	251(0.00%)	282,063 (1.93%)	118,011 (0.75%)	940.21	470.16

**Matched to *A. thaliana *genome**	3,158 (0.05%)	3,269 (0.04%)	7,341 (0.05%)	7,468 (0.04%)	2.32	2.28

**Other sRNAs**	6,682,711 (90.01%)	7,842,554 (99.36%)	13,717,176 (93.76%)	15,035,793 (96.12%)	2.05	1.92

**Total**	6,748,998 (100%)	7,894,661 (100%)	14,630,881 (100%)	15,644,334 (100%)	2.17	1.98

### Differentially expressed miRNAs

**Figure 4:**
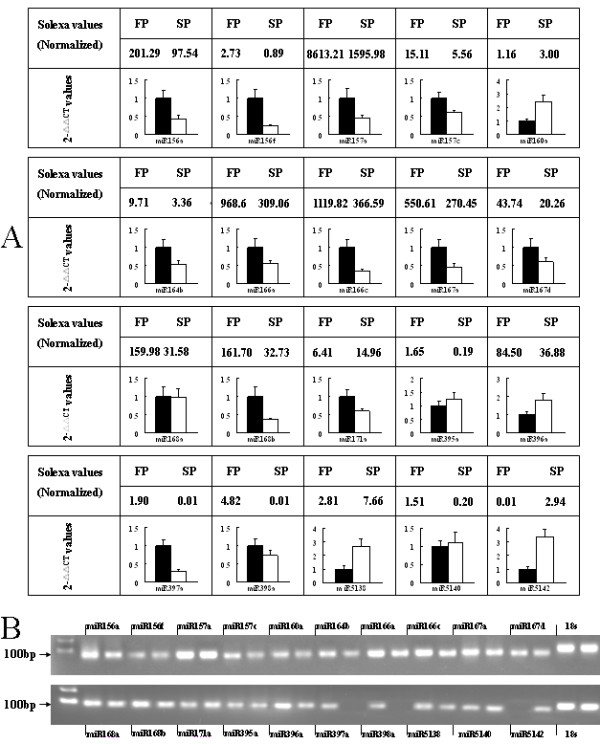
**Comparison of partial miRNAs expressed levels between FP and SP using different methods**. (A) Solexa sequencing (normalized values) and qRT-PCR. (B) Electrophoresis of the qRT-PCR products (FP in left and SP in right).

**Figure 5:**
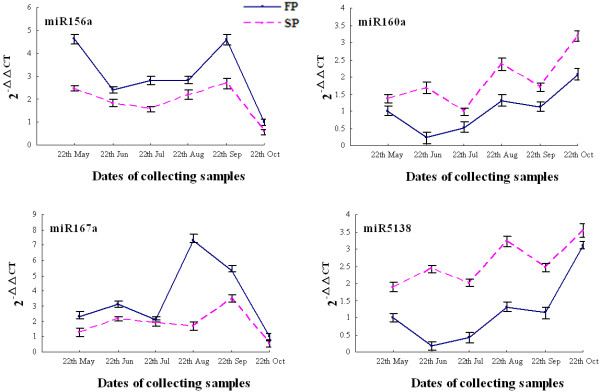
**Differential expression levels of 4 miRNAs in roots**.

Evidence for differential expression in FP and SP plants was sought by comparing the frequency of occurrence of the 89 conserved and six novel miRNAs, based on a Poisson distribution approach [37]. The 29 conserved (11 miRNA families) and three novel miRNAs showing the greatest degree of differential expression are listed in Table 3. Of these 32 sequences, 12 showed a greater than two fold difference in expression level between FP and SP plants. Seven of them were more strongly expressed in SP than in FP plants. The expression levels of the most differentially expressed (17 conserved and 3 novel) miRNAs were reanalysed using qRT-PCR. This confirmed that 14 of the former and two of the latter sequences were indeed differentially expressed in FP and SP plants (Figure 4), showing that frequency of occurrence in Solexa runs produces a reasonably accurate prediction for expression level. Expression levels of 4 miRNAs (miR157a, miR167a, miR160a and miR5138) in roots were measured in different times (Figure 5). miR157a and miR167a were highly expressed in FP, while miR160a and miR5138 were quite opposite, with strongly expressing in SP.

**Table 3:** miRNAs expressed differentially in FP and SP plants.

miRNAs	Sequencing frequency		Normalized value		Fold-change **(log**_**2**_^**SP/FP**^	P-value	Significance
				
	FP	SP	FP	SP			
rgl-miR156a	2,945	1,526	201.29	97.54	-1.05	0.00	**
rgl-miR156b	63	30	4.31	1.92	-1.17	0.00	**
rgl-miR156f	40	14	2.73	0.89	-1.61	0.00	**
rgl-miR157a	126,264	24,968	8,613.21	1,595.98	-2.43	3.45E-263	**
rgl-miR157b	2,154	1,071	147.22	68.46	-1.1	4.40E-05	**
rgl-miR157c	221	87	15.11	5.56	-1.44	1.26E-11	**
rgl-miR157d	158	53	10.8	3.39	-1.67	0.00	**
rgl-miR160a	17	47	1.16	3	1.37	7.87E-16	**
rgl-miR160b	2	26	0.14	1.66	3.6	0.00	**
rgl-miR160c	3	32	0.21	2.05	3.32	0.00	**
rgl-miR164b	153	62	9.71	3.96	-1.29	0.00	**
rgl-miR166a	15,257	4,835	968.6	309.06	-1.65	0.00	**
rgl-miR166b	15,607	4,964	990.82	317.3	-1.64	3.65E-66	**
rgl-miR166c	17,639	5,735	1,119.82	366.59	-1.61	0.00	**
rgl-miR167a	8,673	4,231	550.61	270.45	-1.03	0.00	**
rgl-miR167b	8,164	3,965	518.29	253.45	-1.03	0.00	**
rgl-miR167d	689	317	43.74	20.26	-1.11	0.00	**
rgl-miR168a	2,520	494	159.98	31.58	-2.34	0.00	**
rgl-miR168b	2,547	512	161.70	32.73	-2.3	0.00	**
rgl-miR171a	101	234	6.41	14.96	1.22	2.82E-59	**
rgl-miR171b	37	81	2.35	5.18	1.14	0.00	**
rgl-miR395a	26	3	1.65	0.19	-3.11	0.00	**
rgl-miR395b	11	0	0.7	0.06	-3.45	0.00	**
rgl-miR396a	1,331	577	84.5	36.88	-1.2	0.00	**
rgl-miR396b	656	295	41.65	18.86	-1.14	0.00	**
rgl-miR396c	451	221	28.63	14.13	-1.02	0.00	**
rgl-miR397a	30	0	1.9	0.01	-7.57	0.00	**
rgl-miR398a	76	0	4.82	0.01	-8.91	8.50E-36	**
rgl-miR398b	37	0	2.35	0.01	-7.88	3.24E-10	**
rgl-miR5138	32	84	2.81	7.67	1.44	0.00	**
rgl-miR5140	39	5	1.51	0.20	-2.96	0.00	**
rgl-miR5142	0	30	0.01	2.94	8.2	0.00	**

### Target prediction for the three differentially expressed novel miRNAs

The target of most plant miRNAs possesses a single perfect or near perfect complementary site in the coding region [13,15]. Assuming this to be generally the case, the *A. thaliana *gene space was searched for complementarity with the sequences of the three differentially expressed novel miRNAs. Using a set of rules for predicting novel miRNA potential target genes [14,38], this exercise predicted seven potential targets, with miR5138 and miR5140 both targeting more than one gene (Table S3 in Additional file 1). The targets encoded the following gene products: ICU2 (INCURVATA2), a DNA-directed DNA polymerase, a magnesium transporter CorA-like family protein, an ATP synthase (α chain), a TIR-NBS-LRR protein, a ZIGA4 (ARF GAP-like zinc finger-containing protein ZiGA4) and a DC1 domain-containing protein.

### Function of the potential targets of differentially expressed miRNAs

**Figure 6:**
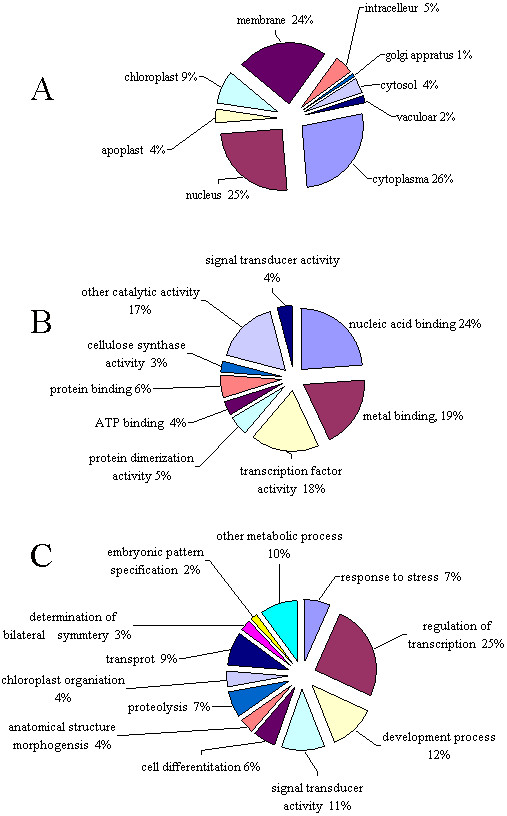
**Gene ontology of the predicted targets for 32 differentially expressed miRNAs**. Categorization of miRNA-target genes was performed according to the cellular component (A), molecular function (B) and biological process (C).

An indication of the genes responsible for the continuous cropping syndrome was sought by an inspection of the 308 potential targets of the 29 differentially expressed miRNAs (Additional file 3) in addition to the seven targets of the novel miRNAs (Table S3 in Additional file 1). Gene ontology categories were assigned to all 315 putative targets according to their cellular component, their molecular function and the biological process(es) they are involved in *A. thaliana *(Figure 6). With respect to molecular function, the targets fell largely into nine categories, with the three most over-represented being nucleic acid binding, metal ion binding and transcription factor activity. Twelve biological processes were identified, with the three most frequent being the regulation of transcription, plant development and signal transduction. The potential targets for the 32 differentially expressed miRNAs mainly involved transcription, plant development and signal transduction.

**Figure 7:**
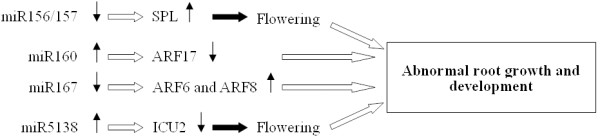
**Possible functions between differentially expressed miRNAs and their targets in growth and development of *R. glutinosa***. ↑: up- regulation of expression, ↓: down-regulation of expression. Empty arrows imply inhibition of phenotype, while solid arrows indicate its promotion.

**Figure 8:**
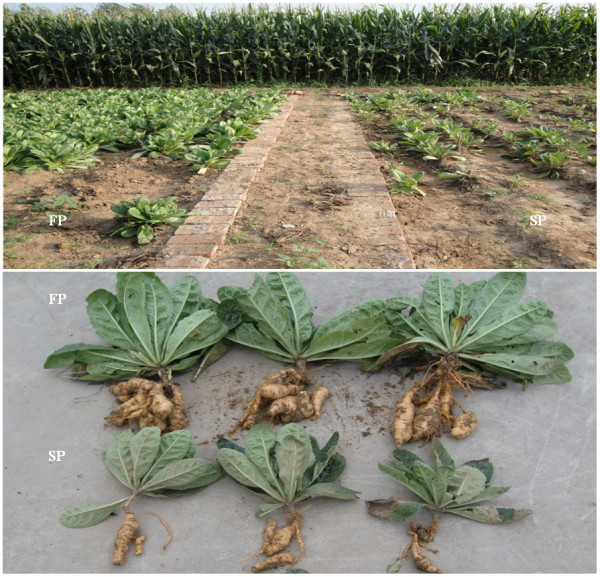
**Difference of FP and SP *R. glutinosa *plants**.

Several of these targets may be directly or indirectly involved in the development of tuberous vs fibrous roots (Figure 7, 8). For example, miR156/157 targets an SPL transcription factor, which when over-expressed in *A. thaliana*, produces an early flowering phenotype. The over-expression of miR156/157 itself delays flowering [39-41]. Thus it is possible that in *R. glutinosa*, a higher level of expression of miR156/157 (as occurred in FP plants) could prolong root growth and development. The miR160 target is the auxin response factor ARF17, while those of miR167 are ARF6 and ARF8. ARF17 is a negative regulator, while ARF6 and ARF8 are positive regulators of adventitious rooting. These three ARFs share overlapping expression domains, interact genetically and regulate one another's expression at both the transcriptional and post-transcriptional level [42]. Since SP plants express more miR160 and less miR167 than FP plants, it is possible that the balance of ARF protein present is altered by continuous cropping, and hence there is an effect on tuberous root expansion. The target of miR5138 is the gene *ICU2*, which is negative regulator of floral homeotic genes in *A. thaliana*. Its over-expression delays flowering, while its knock-out hastens it [43]. Since this miRNA is more highly expressed in SP than in FP plants, there may be a differential expression of *ICU2 *and hence an effect on flowering time, with a knock-on effect on tuberous root expansion.

These predicted target genes were cloned in *R. glutinosa *(Table 4 and Additional file 4) and registered as ESTs in NCBI.

**Table 4:** Partial targets cloned in *R. glutinosa*.

miRNAs	Target Acc. of *A.thaliana*	Genbank Acc. of targets cloned in	Identity	Annotation (*A. thaliana*)
rgl-miR160	AT4G30080.1	JG014346	79.94%	ARF16 (Auxin response factor 16)
rgl-miR167	AT1G30330.1	JG390498	67.51%	ARF6 (Auxin response factor 6)
rgl-miR5138	AT5G67100.1	JG390599	76.68%	ICU2 (INCURVATA2); DNA-directed DNA polymerase
rgl-miR5140	AT3G58970.1	JG390538	68.87%	magnesium transporter CorA-like family protein

Overall, there was a suggestion that the expression of a number of miRNA families may be correlated with the continuous cropping syndrome in *R. glutinosa*. Whether these miRNAs actually regulate key genes responsible for the syndrome will require experimental demonstration. The identification of these miRNAs has nevertheless succeeded in providing leads for determining the molecular genetic basis of the continuous cropping syndrome in *R. glutinosa*.

## Conclusions

Here we have described the application of a combination of approaches to identify a set of 89 conserved (belonging to 25 families) and six novel *R. glutinosa *miRNAs, which are differentially, expressed in first and second year crops. We believe that this information could provide initial candidates for the genes responsible for tuberous root expansion, and in particular for the syndrome of continuous cropping yield decline in this medicinally important species.

## Methods

### Plant material and RNA isolation

*R. glutinosa *cultivar "Wen 85-5" was collected from the Wen Agricultural Institute, Jiaozuo City, Henan Province, China. The first year crop (FP) was grown from April 15 to November 30 2009, and the second year crop (SP) was planted on the same date, but on land where a first crop had been grown the previous year (plant growth period was between April 15, 2008 and November 30, 2008) (Figure 8). Leaf, stem and root samples were taken from five independent plants at the tuberous root expansion stage (August 15, 2009), and their RNA content was extracted with the TriZOL reagent (TaKaRa Co., Tokyo, Japan). Total RNA from each plant was pooled, and then separated by 15% denaturing PAGE to recover the population of small RNAs (size range 18-30 nt) present.

For the measure of differently expressed miRNAs in various development stages of *R. glutinosa*, FP and SP plants (cultivar "Wen 85-5") were grown in the isolated plots from April 22 to October 22, 2010. Roots of *R. glutinosa *were collected every month and total RNAs were extracted with TriZOL reagent.

### miRNA library construction and sequencing

The small RNAs were ligated sequentially to 5' and 3' RNA/DNA chimeric oligonucleotide adaptors (Illumina), and the resulting ligation products were gel purified by 10% denaturing PAGE, and reverse transcribed. The cDNAs obtained in this way were sequenced on a Genome Analyzer IIx System by Beijing Genomics Institute (BGI) (Shenzhen, China).

### Identification of miRNAs

Conserved miRNAs were identified by blastn searches against Genbank http://www.ncbi.nlm.nih.gov, Rfam 9.1 (rfam.janelia.org) and miRBase 15.0 http://www.mirbase.org databases with default parameters. Potentially novel sequences were identified by an alignment with the *A. thaliana *genome sequence ftp://ftp.tigr.org/pub/data/a_thaliana/ath1/SEQUENCES/ using SOAP (soap.genomics.org.cn) software [44]. Candidate pre-miRNAs were identified by folding the flanking genome sequence of distinct miRNAs using MIREAP (mireap.sourceforge.net), followed by a prediction of secondary structure by mFold v3.1 [45]. The criteria chosen for stem-loop hairpins were as suggested elsewhere [35,36].

### Reverse transcription (RT) reaction

For RT, polyA was first added to the 3' end of the miRNAs using polyA polymerase, and cDNA was then synthesized using AMV reverse transcriptase (GeneCopoeia, Inc.), employing a 53 nt oligodT-adaptor sequence (GeneCopoeia, Inc.) as the primer. The former was a 25 μl reaction, containing 2 μg total RNA, 2.5U polyA polymerase (GeneCopoeia, Inc.), 1 μl RTase mixture (GeneCopoeia, Inc.), and 5 μl 5× reaction buffer. The reaction was incubated at 37°C for 60 min and 85°C for 5 min, and then stored at -20°C.

### Identification of novel miRNAs using RT-PCR

Forward primers (sequence given in Table S4 in Additional file 1) were synthesized by Sangon (Shanghai, China). Each 50 μl reaction comprised 0.5 μl cDNA, 2 μl (2 μM) miRNA forward primer, 2 μl (2 μM) reverse primer (Universal Adaptor PCR Primer, GeneCopoeia, Inc.), 5 μl 10× PCR buffer, 2 μl 10 mM dNTP, 1U Taq DNA polymerase (Invitrogen, Inc.). The reactions were initially denatured at 95°C for 10 min, and then cycled 36 times through 95°C/10 s, 55°C/20 s, 72°C/10 s. A 5 μl aliquot of each reaction was subjected to 3% agarose electrophoresis.

### Validation of differential miRNA expression based on qRT-PCR

qRT-PCR was performed using an All-in-One™ miRNA Q-PCR detection kit (GeneCopoeia, Inc.) on a BIO-RAD iQ5 real-time PCR detection system (Bio-Rad laboratories, Inc.). Each 20 μl Q-PCR comprised 0.5 μl cDNA, 2 μl 2 μM miRNA forward primer (sequence given in Table S5 in Additional file 1), 2 μl 2 μM reverse primer (Universal Adaptor PCR Primer), 10 μl 2× All-in-One™ miRNA Q-PCR buffer and 5.5 μl nuclease-free water. The reactions were incubated at 95°C for 10 min, then were cycled 36 times through 95°C/10 s, 55°C/20 s and 72°C/10 s. After the reactions had been completed, the threshold was manually set and the threshold cycle (CT) was automatically recorded. All reactions were replicated twice per biological sample. A 4 μl aliquot of each reaction product was subjected to 3% agarose electrophoresis. The relative expression level of the miRNAs was calculated using the 2 ^-ΔΔCT ^method [46], and the data were normalized on the basis of 18 s rRNA CT values.

### Target gene prediction and annotation of novel miRNAs

Potential targets of novel miRNAs were predicted *in silico *a software package developed by the Huada Genomic Center (Beijing, China, http://www.rnaiweb.com/RNAi/MicroRNA/MicroRNA_Tools___Software/MicroRNA_Target_Scan/index.html) mounted in the *A. thaliana *transcript database ftp://ftp.tigr.org/pub/data/a_thaliana/ath1/SEQUENCES/. The criteria applied were as described elsewhere [14,38]. The potential targets of conserved miRNA families were identified by a search in the website http://bioinfo3.noble.org/psRNATarget/, with the following settings applied: transcript/genomic library *A. thaliana *TAIR7 cDNA [25/04/2007 release]; range of maximum expectation 1-5; range of maximum circles 1-3; range of central mismatch for translational inhibition 9-11 nt. A BlastN search against a reference *A. thaliana *database including UniProt entries http://www.uniprot.org/ was used to provide gene ontologies, expressed as three independent hierarchies: biological process, cell component and molecular function.

## Authors' contributions

YY carried out *R. glutinosa *small RNA isolation, participated in the computational analyses and drafted manuscript. XC participated in the bioinformatics analyses and drafted manuscript. JC carried out the molecular genetic studies. HX participated in the sequence alignment. JL carried out field *R. glutinosa *plant cultivation and collection. ZZ conceived of the study, participated in its design and drafted and amended the manuscript. All authors read and approved the final manuscript.

## Supplementary Material

Additional file 1 **Table S1 - Conserved miRNAs from *R. glutinosa***. The abbreviations represent: ath, *A. thaliana*; gma, soybean; ptc, black poplar; vvi, grape; osa, rice. The plus symbols indicate: ++, miRNA sequences of *R*. *glutinosa *were exactly identical to those in other species; +, miRNA sequences of *R. glutinosa *were conserved in other species but have variations in some nucleotide positions. Table S2 - Candidates of novel miRNAs from *R. glutinosa*. Table S3 - Predicted targets of novel validated *R. glutinosa *miRNAs. Table S4 - Forward primer sequences of candicate miRNAs using RT-PCR in *R. glutinosa*. Click here for file

Additional file 2 **Secondary structures of candidate miRNAs**. Click here for file

Additional file 3 **Potential target genes of 29 conserved miRNAs**. Click here for file

Additional file 4 **Sequence alignments of partial targets of differential expressed miRNAs**. Click here for file
