# Supplementary material for: Differential miRNA expression in Rehmannia glutinosa plants subjected to continuous cropping
Source: BMC Plant Biol. 2011 Mar 26;11:53. doi: 10.1186/1471-2229-11-53 (PMC3078876; doi:10.1186/1471-2229-11-53)
Supplement: Additional file 1 — Table S1 - Conserved miRNAs from R. glutinosa. The abbreviations represent: ath, A. thaliana; gma, soybean; ptc, black poplar; vvi, grape; osa, rice. The plus symbols indicate: ++, miRNA sequences of R. glutinosa were exactly identical to those in other species; +, miRNA sequences of R. glutinosa were conserved in other species but have variations in some nucleotide positions. Table S2 - Candidates of novel miRNAs from R. glutinosa. Table S3 - Predicted targets of novel validated R. glutinosa miRNAs. Table S4 - Forward primer sequences of candicate miRNAs using RT-PCR in R. glutinosa. [file 1471-2229-11-53-S1.DOC]

**Additional file 1**

**Table S1 - Conserved miRNAs from *R. glutinosa*.**

| **miRNA family** | **miRNA family** | **Sequence (5' - 3')** | **Length** | **Conserved in** | | | | | **Reads** | |
| --- | --- | --- | --- | --- | --- | --- | --- | --- | --- | --- |
|  |  |  | **(nt)** | **ath** | **gma** | **ptc** | **vvi** | **osa** | **FP** | **SP** |
| 156/157 | rgl-miR156 |  |  | ++ | ++ | ++ | ++ | ++ | 3,076 | 1,583 |
|  | rgl-miR156a | UGACAGAAGAGAGUGAGCAC | 20 | ++ | ++ | ++ | ++ | ++ | 2,945 | 1,526 |
|  | rgl-miR156b | UGACAGAAGAGAGUGAGCACA | 21 | + | + | + | + | + | 63 | 30 |
|  | rgl-miR156c | CUGACAGAAGAGAGUGAGCAC | 21 | + | + | + | + | + | 21 | 10 |
|  | rgl-miR156d | ACUGACAGAAGAGAGUGAGC | 20 | + | + | + | + | + | 2 | 2 |
|  | rgl-miR156e | CGACAGAAGAGAGUGAGCAC | 20 | + | + | + | + | + | 4 | 1 |
|  | rgl-miR156f | UUGACAGAAGAGAGUGAGCAC | 21 | + | + | + | + | + | 40 | 14 |
|  | rgl-miR157 |  |  |  |  |  |  |  | 128,797 | 26,179 |
|  | rgl-miR157a | UUGACAGAAGAUAGAGAGCAC | 21 | ++ | ++ | ++ | + | + | 126,264 | 24,968 |
|  | rgl-miR157b | UGACAGAAGAUAGAGAGCAC | 20 | ++ | ++ | ++ | ++ | + | 2,154 | 1,071 |
|  | rgl-miR157c | UUGACAGAAGAUAGAGAGCACA | 22 | ++ | ++ | ++ | ++ | + | 221 | 87 |
|  | rgl-miR157d | UGACAGAAGAUAGAGAGCACA | 21 | ++ | ++ | ++ | ++ | + | 158 | 53 |
| 158 | rgl-miR158a | UCCCAAAUGUAGACAAAGCA | 20 | ++ |  |  |  |  | 8 |  |
| 159/319 | rgl-miR159 |  |  |  |  |  |  |  | 3,849 | 6,276 |
|  | rgl-miR159a | UUUGGAUUGAAGGGAGCUCUA | 21 | ++ | ++ | ++ | ++ | + | 3,643 | 6,017 |
|  | rgl-miR159b | UUUGGAUUGAAGGGAGCUCUU | 21 | ++ | + | + | + | + | 121 | 203 |
|  | rgl-miR159c | UUUGGAUUGAAGGGAGCUCCU | 21 | ++ | + | + | + | + | 85 | 56 |
|  | rgl-miR319 |  |  |  |  |  |  |  | 34 | 24 |
|  | rgl-miR319a | UUGGACUGAAGGGAGCUCCCU | 21 | ++ | + | + | ++ | + | 18 | 14 |
|  | rgl-miR319b | UGCUUGGACUGAAGGGAGCUC | 21 | + | + | + | + | + | 14 | 9 |
|  | rgl-miR319c | UUGGACUGAAGGGAGCUCCU | 20 | ++ | + | + | + | + | 2 | 1 |
| 160 | rgl-miR160 |  |  |  |  |  |  |  | 22 | 99 |
|  | rgl-miR160a | UGCCUGGCUCCCUGUAUGCCA | 21 | ++ | ++ | ++ | ++ | ++ | 17 | 47 |
|  | rgl-miR160b | AUGCCUGGCUCCCUGUAUGCC | 21 | ++ | ++ | ++ | ++ | ++ | 2 | 26 |
|  | rgl-miR160c | GCCUGGCUCCCUGUAUGCCA | 20 | ++ | ++ | ++ | ++ | ++ | 3 | 32 |
| 161 | rgl-miR161 | UUGAAAGUGACUACAUCGGGG | 21 | ++ |  |  |  |  | 1 | 0 |
| 164 | rgl-miR164 |  |  |  |  |  |  |  | 9,942 | 7,464 |
|  | rgl-miR164a | UGGAGAAGCAGGGCACGUGCA | 21 | ++ | ++ | ++ | ++ | ++ | 9,713 | 7,344 |
|  | rgl-miR164b | UGGAGAAGCAGGGCACGUGCG | 21 | ++ | ++ | ++ | ++ | ++ | 153 | 62 |
|  | rgl-miR164c | GGAGAAGCAGGGCACGUGCAA | 21 | ++ | ++ | ++ | ++ | ++ | 76 | 59 |
| 165/166 | rgl-miR165 |  |  |  |  |  |  |  | 31 | 18 |
|  | rgl-miR165a | UCGGACCAGGCUUCAUCCCCC | 21 | ++ | ++ | + | + | + | 28 | 17 |
|  | rgl-miR165b | UCGGACCAGGCUUCAUCCCC | 20 | ++ | ++ | + | + | + | 3 | 1 |
|  | rgl-miR166 |  |  |  |  |  |  |  | 48,740 | 15,731 |
|  | rgl-miR166a | UCGGACCAGGCUUCAUUCCC | 20 | ++ | ++ | ++ | ++ | ++ | 15,257 | 4,835 |
|  | rgl-miR166b | UUCGGACCAGGCUUCAUUCC | 20 | ++ | ++ | ++ | ++ | ++ | 15,607 | 4,964 |
|  | rgl-miR166c | CGGACCAGGCUUCAUUCCCC | 20 | ++ | ++ | ++ | ++ | ++ | 17,639 | 5,735 |
|  | rgl-miR166d | CUUCGGACCAGGCUUCAUUCC | 21 | + | + | + | + | + | 156 | 130 |
|  | rgl-miR166e | CGGACCAGGCUUCAUUCCCCC | 21 | ++ | ++ | ++ | ++ | ++ | 43 | 37 |
|  | rgl-miR166f | UCGGACCAGGCUUCAUUCCCC | 21 | ++ | ++ | ++ | ++ | ++ | 35 | 30 |
|  | rgl-miR166g | UUCGGACCAGGCUUCAUUCCC | 21 | + | + | + | + | + | 3 | 0 |
| 167 | rgl-miR167 |  |  |  |  |  |  |  | 17,533 | 8,513 |
|  | rgl-miR167a | UGAAGCUGCCAGCAUGAUCUA | 21 | ++ | ++ | ++ | ++ | ++ | 8,673 | 4,231 |
|  | rgl-miR167b | GAAGCUGCCAGCAUGAUCUAA | 21 | ++ | ++ | ++ | ++ | ++ | 8,164 | 3,965 |
|  | rgl-miR167c | GAAGCUGCCAGCAUGAUCUAU | 21 | + | + | + | + | + | 7 | 3 |
|  | rgl-miR167d | UGAAGCUGCCAGCAUGAUCUGG | 22 | + | + | + | + | + | 689 | 317 |
| 168 | rgl-miR168 |  |  |  |  |  |  |  | 5,079 | 1,015 |
|  | rgl-miR168a | UCGCUUGGUGCAGGUCGGGAA | 21 | ++ | ++ | ++ | ++ | + | 2,520 | 494 |
|  | rgl-miR168b | UUCGCUUGGUGCAGGUCGGGA | 21 | ++ | ++ | ++ | ++ | + | 2,547 | 512 |
|  | rgl-miR168c | CGCUUGGUGCAGGUCGGGAAC | 21 | ++ | ++ | ++ | ++ | + | 12 | 9 |
| 169 | rgl-miR169 |  |  |  |  |  |  |  | 107 | 101 |
|  | rgl-miR169a | CAGCCAAGGAUGACUUGCCGA | 21 | ++ | ++ | ++ | ++ | ++ | 52 | 51 |
|  | rgl-miR169b | CAGCCAAGGAUGACUUGCCGG | 21 | ++ | ++ | ++ | ++ | ++ | 16 | 12 |
|  | rgl-miR169c | CAGCCAAGGAUGACUUGCCG | 20 | ++ | ++ | ++ | ++ | ++ | 12 | 11 |
|  | rgl-miR169d | UGAGCCAAGGAUGACUUGCC | 21 | ++ | ++ | ++ | + | + | 3 | 7 |
|  | rgl-miR169e | UGAGCCAAGGAUGACUUGCCG | 21 | ++ | ++ | ++ | + | + | 15 | 13 |
|  | rgl-miR169f | UAGCCAAGGAUGACUUGCCUG | 21 | ++ | ++ | ++ | + | ++ | 6 | 5 |
|  | rgl-miR169h | UAGCCAAGGAUGACUUGCCUGC | 22 | ++ | ++ | ++ | + | ++ | 1 | 0 |
|  | rgl-miR169i | UAGCCAAGGAUGACUUGCC | 19 | ++ | ++ | ++ | + | ++ | 2 | 2 |
| 171 | rgl-miR171 |  |  |  | ++ |  |  |  | 139 | 324 |
|  | rgl-miR171a | UUGAGCCGUGCCAAUAUCAC | 20 | ++ | ++ | ++ | + | + | 101 | 234 |
|  | rgl-miR171b | UUGAGCCGUGCCAAUAUCACG | 21 | ++ | ++ | ++ | + | + | 37 | 81 |
|  | rgl-miR171c | UGAUUGAGCCGCGCCAAUAUC | 21 | ++ | ++ | + | ++ | ++ | 1 | 9 |
| 172 | rgl-miR172 |  |  |  |  |  |  |  | 58,477 | 46,099 |
|  | rgl-miR172a | AGAAUCUUGAUGAUGCUGCAU | 21 | ++ | ++ | ++ | ++ | ++ | 56,376 | 44,434 |
|  | rgl-miR172b | AGAAUCUUGAUGAUGCUGCAG | 21 | ++ | ++ | + | + | + | 897 | 723 |
|  | rgl-miR172c | GGAAUCUUGAUGAUGCUGCAU | 21 | ++ | ++ | ++ | + | + | 1,148 | 897 |
|  | rgl-miR172d | GAAUCUUGAUGAUGCUGCAUC | 21 | ++ | ++ | ++ | ++ | ++ | 13 | 10 |
|  | rgl-miR172e | GAAUCUUGAUGAUGCUGCAGC | 21 | + | + | + | + | + | 43 | 35 |
| 390 | rgl-miR390 |  |  |  |  |  |  |  | 595 | 665 |
|  | rgl-miR390a | AAGCUCAGGAGGGAUAGCGCC | 21 | ++ | ++ | ++ | ++ | ++ | 548 | 575 |
|  | rgl-miR390b | AAGCUCAGGAGGGAUAGCGCC | 21 | ++ | ++ | ++ | ++ | ++ | 19 | 23 |
|  | rgl-miR390c | GCUCAGGAGGGAUAGCGCCAUG | 22 | + | + | + | + | + | 5 | 9 |
|  | rgl-miR390d | CGCUAUCCAUCCUGAGUUUC | 20 | + | ++ | + | + | + | 23 | 30 |
|  | rgl-miR390e | AGCUCAGGAGGGAUAGCGCC | 20 | ++ | ++ | ++ | ++ | ++ | 21 | 28 |
| 393 | rgl-miR393a | AUCAUGCUAUCUCUUUGGAUU | 21 | + |  | + | + | + | 10 | 8 |
| 394 | rgl-miR394 |  |  |  |  |  |  |  | 1,352 | 1,636 |
|  | rgl-miR394a | UUGGCAUUCUGUCCACCUCC | 20 | ++ |  | ++ | ++ | ++ | 1,313 | 1,601 |
|  | rgl-miR394b | UUUGGCAUUCUGUCCACCUCC | 21 | ++ |  | ++ | ++ | ++ | 13 | 12 |
|  | rgl-miR394c | UGGCAUUCUGUCCACCUCCU | 20 | + |  | + | + | + | 26 | 23 |
| 395 | rgl-miR395 |  |  |  |  |  |  |  | 37 | 3 |
|  | rgl-miR395a | CUGAAGUGUUUGGGGGAACUC | 21 | ++ |  | ++ | ++ | + | 26 | 3 |
|  | rgl-miR395b | UGAAGUGUUUGGGGGAACUC | 20 | ++ |  | ++ | ++ | + | 11 | 0 |
| 396 | rgl-miR396 |  |  |  |  |  |  |  | 2,449 | 1,102 |
|  | rgl-miR396a | GUUCAAUAAAGCUGUGGGAAG | 21 | ++ | + | ++ | ++ | ++ | 1,331 | 577 |
|  | rgl-miR396b | UUCCACAGCUUUCUUGAACUU | 21 | + | ++ | ++ | + | ++ | 656 | 295 |
|  | rgl-miR396c | UUCCACAGCUUUCUUGAACUG | 21 | ++ | ++ | ++ | ++ | ++ | 451 | 221 |
|  | rgl-miR396d | UCUUCCACAGCUUUCUUGAACU | 22 | + | + | + | + | + | 2 | 2 |
|  | rgl-miR396e | UCAAUAAAGCUGUGGGAAGAUA | 22 | + | + | + | + | + | 8 | 7 |
|  | rgl-miR396f | ACAGCUUUCUUGAACUGCAAA | 21 | + | + | + | + | + | 1 | 0 |
| 397 | rgl-miR397a | UCAUUGAGUGCAGCGUUGAUG | 21 | ++ |  | ++ | ++ | ++ | 30 | 0 |
| 398 | rgl-miR398 |  |  |  |  |  |  |  | 117 | 0 |
|  | rgl-miR398a | UGUGUUCUCAGGUCACCCCUU | 21 | ++ | ++ | + | + | + | 76 | 0 |
|  | rgl-miR398b | UGUGUUCUCAGGUCACCCCUG | 21 | ++ | + | + | + | + | 37 | 0 |
|  | rgl-miR398c | UUGUGUUCUCAGGUCACCCCUU | 22 | + | + | + | + | + | 1 | 0 |
|  | rgl-miR398d | UGUUCUCAGGUCACCCCUUUGA | 22 | + | + | + | + | + | 1 | 0 |
|  | rgl-miR398e | GUGUUCUCAGGUCACCCCUG | 20 | ++ | + | + | + | + | 2 | 0 |
| 399 | rgl-miR399 |  |  |  |  |  |  |  | 6 | 3 |
|  | rgl-miR399a | UGCCAAAGGAGAGUUGCCCUG | 21 | ++ |  | + | ++ | ++ | 3 | 2 |
|  | rgl-miR399b | UGCCAAAGGAGAGUUGCCCU | 20 | ++ |  | + | ++ | ++ | 2 | 0 |
|  | rgl-miR399c | UGCCAAAGGAGAUUUGCCCGG | 21 | ++ |  | ++ | ++ | + | 1 | 1 |
| 403 | rgl-miR403 |  |  | ++ |  | ++ | ++ |  | 1,818 | 1,427 |
|  | rgl-miR403a | UUAGAUUCACGCACAAACUCG | 21 | ++ |  | ++ | ++ |  | 1,817 | 1,427 |
|  | rgl-miR403b | UUAGAUUCACGCACAAACUCGU | 22 | + |  | + | + |  | 1 | 0 |
| 408 | rgl-miR408 | AUGCACUGCCUCUUCCCUGGC | 21 | ++ |  | ++ | ++ | + | 10 | 0 |
| 822 | rgl-miR822 | UGCGGGAAGCAUUUGCACAUG | 21 | ++ |  |  |  |  | 2 | 0 |
| 858 | rgl-miR858 |  |  |  |  |  |  |  | 91 | 136 |
|  | rgl-miR858a | UUUCGUUGUCUGUUCGACCU | 20 | ++ |  |  |  |  | 19 | 30 |
|  | rgl-miR858b | UUCGUUGUCUGUUCGACCUUG | 21 | ++ |  |  |  |  | 72 | 106 |
| 2111 | rgl-miR2111 |  |  |  |  |  |  |  | 11 | 1 |
|  | rgl-miR2111a | UAAUCUGCAUCCUGAGGUUUA | 21 | ++ |  |  |  |  | 6 | 1 |
|  | rgl-miR2111b | UAAUCUGCAUCCUGAGGUUU | 20 | ++ |  |  |  |  | 5 | 0 |
| The abbreviations represent: ath, *A. thaliana*; gma, soybean; ptc, black poplar; vvi, grape; osa, rice. The plus symbols indicate: ++, miRNA sequences of R. *glutinosa* were exactly identical to those in other species; +, miRNA sequences of R*. glutinosa* were conserved in other species but have variations in some nucleotide positions. | | | | | | | | | | |
|  |  |  |  |  |  |  |  |  |  |  |
|  |  |  |  |  |  |  |  |  |  |  |
|  |  |  |  |  |  |  |  |  |  |  |

Table S2 - Candidates of novel miRNAs from *R. glutinosa*.

| **sRNA** | **Reads number** | | **Sequence** | **Energy** | **RT-PCR** | |
| --- | --- | --- | --- | --- | --- | --- |
|  | **FP** | **SP** |  | **(kcal mol^-1^)** | FP | SP |
| rgl-miR5137 | 10 | 8 | AGCGGAGAAGACGAUGGGCU | -25.7 | Yes | Yes |
| Z2 | 38 | 15 | UUUAUAUGUUAUUUUUUG | -26.33 | No | No |
| rgl-miR5138 | 32 | 84 | AAAAAUCGUUAGGCGCUA | -29.5 | Yes | Yes |
| Z4 | 13 | 7 | UUUGUGCAAAAUUUUAGAA | -28.2 | No | No |
| Z5 | 10 | 6 | CUUCUAAAGGAAGUCUUU | -20.2 | No | No |
| Z6 | 39 | 30 | GUUCGUGUAUUUGUCAUC | -44.6 | No | No |
| rgl-miR5139 | 17 | 9 | AAACCUGGCUCUGAUACCA | -26.79 | Yes | Yes |
| Z8 | 7 | 3 | GAUGAUGAUGAUGACGACGACGAC | -36.9 | No | No |
| Z9 | 5 | 4 | GUUUAUUUUUGAUAAAAA | -20.5 | No | No |
| rgl-miR5140 | 39 | 5 | GCUGGUGAAGAUUUGGUG | -45.6 | Yes | Yes |
| Z11 | 5 | 3 | CAAUGUUUUAUAUAAGUU | -26.9 | No | No |
| Z12 | 17 | 8 | AGGGAUGGAGGAUAUGCU | -32.7 | No | No |
| Z13 | 10 | 7 | GAUUAAUCGGGCUAGGCGGAGAU | -33.22 | No | No |
| Z14 | 16 | 5 | UGUGGGAUGUGAGAAGAG | -36.2 | No | No |
| rgl-miR5141 | 27 | 19 | AGACCCGACGCGACUGACAGAUAA | -36.5 | Yes | Yes |
| Z16 | 5 | 6 | AGACAGAAGAACAUGCCU | -29.6 | No | No |
| Z17 | 7 | 4 | ACCUUGUGAGGAACUCAGAGAUGG | -38.6 | No | No |
| rgl-miR5142 | 0 | 30 | AUAUUGAUUGAUAAGUGAU | -27.04 | No | Yes |

Table S3 - Predicted targets of novel validated *R. glutinosa* miRNAs.

| **sRNA ID** | **Target gene accession** | **Start-end position of target** | **Target description** |
| --- | --- | --- | --- |
| rgl-miR5138 | AT5G67100.1 | chr5:26776994-26785104 | ICU2 (INCURVATA2); DNA-directed DNA polymerase |
|  | AT2G24560.1 | chr2:10431537-10432933 | carboxylesterase/ hydrolase, acting on ester bonds |
| rgl-miR5140 | AT3G58970.1 | chr3:21789659-21791163 | magnesium transporter CorA-like family protein |
|  | AT1G61590.1 | chr1:22723691-22726022 | protein kinase, putative |
|  | AT5G03860.1 | chr5:1032276-1034527 | MLS (MALATE SYNTHASE); malate synthase |
|  | AT1G59780.1 | chr1:21993581-21997691 | disease resistance protein (CC-NBS-LRR class) |
|  | AT1G08680.1 | chr1:2762820-2768387 | ZIGA4 (ARF GAP-like zinc finger-containing protein ZiGA4) |
|  | AT3G51570.1 | chr3:19126358-19130456 | disease resistance protein (TIR-NBS-LRR class) |
| rgl-miR5142 | AT5G42840.1 | chr5:17179158-17181173 | DC1 domain-containing protein |

Table S4 - Forward primer sequences of candicate miRNAs using RT-PCR in *R. glutinosa*.

| **miRNA** | **Primer sequence** |
| --- | --- |
| rgl-miR5137 | AGCGGAGAAGACGAUGGGCU |
| Z2 | TTTATATGTTATTTTTTG |
| rgl-miR5138 | AAAAATCGTTAGGCGCTA |
| Z4 | TTTGTGCAAAATTTTAGAA |
| Z5 | CTTCTAAAGGAAGTCTTT |
| Z6 | GTTCGTGTATTTGTCATC |
| rgl- miR5139 | AAACCTGGCTCTGATACCA |
| Z8 | GATGATGATGATGACGACGACGAC |
| Z9 | GTTTATTTTTGATAAAAA |
| rgl-miR5140 | GCTGGTGAAGATTTGGTG |
| Z11 | CAATGTTTTATATAAGTT |
| Z12 | AGGGATGGAGGATATGCT |
| Z13 | GATTAATCGGGCTAGGCGGAGAT |
| Z14 | TGTGGGATGTGAGAAGAG |
| rgl-miR5141 | AGACCCGACGCGACTGACAGATAA |
| Z16 | AGACAGAAGAACATGCCT |
| Z17 | ACCTTGTGAGGAACTCAGAGATGG |
| rgl-miR5142 | ATATTGATTGATAAGTGAT |

Table S5 - Forward primer sequences of qRT-PCR validated miRNAs from *R. glutinosa*.

| **miRNA** | **Primer sequence** |
| --- | --- |
| rgl-miR156a | TGACAGAAGAGAGTGAGCAC |
| rgl-miR156f | TTGACAGAAGAGAGTGAGCAC |
| rgl-miR157a | TTGACAGAAGATAGAGAGCAC |
| rgl-miR157c | TTGACAGAAGATAGAGAGCACA |
| rgl-miR160a | TGCCTGGCTCCCTGTATGCCA |
| rgl-miR166a | TCGGACCAGGCTTCATTCCC |
| rgl-miR166c | CGGACCAGGCTTCATTCCCC |
| rgl-miR166e | CGGACCAGGCTTCATTCCCCC |
| rgl-miR167a | TGAAGCTGCCAGCATGATCTA |
| rgl-miR167d | TGAAGCTGCCAGCATGATCTGG |
| rgl-miR168a | TCGCTTGGTGCAGGTCGGGAA |
| rgl-miR168b | TTCGCTTGGTGCAGGTCGGGA |
| rgl-miR171a | TTGAGCCGTGCCAATATCAC |
| rgl-miR395a | CTGAAGTGTTTGGGGGAACTC |
| rgl-miR396a | GTTCAATAAAGCTGTGGGAAG |
| rgl-miR397a | TCATTGAGTGCAGCGTTGATG |
| rgl-miR398a | TGTGTTCTCAGGTCACCCCTT |
| rgl-miR5138 | AAAAATCGTTAGGCGCTA |
| rgl-miR5140 | CTGGTGAAGATTTGGTGG |
| rgl-miR5142 | ATATTGATTGATAAGTGAT |
