# Supplementary material for: Differential miRNA expression in Rehmannia glutinosa plants subjected to continuous cropping
Source: BMC Plant Biol. 2011 Mar 26;11:53. doi: 10.1186/1471-2229-11-53 (PMC3078876; doi:10.1186/1471-2229-11-53)

### Additional file 2 – Secondary structures of candidate miRNAs

Secondary structure for ‘rgl-miR5137**’**


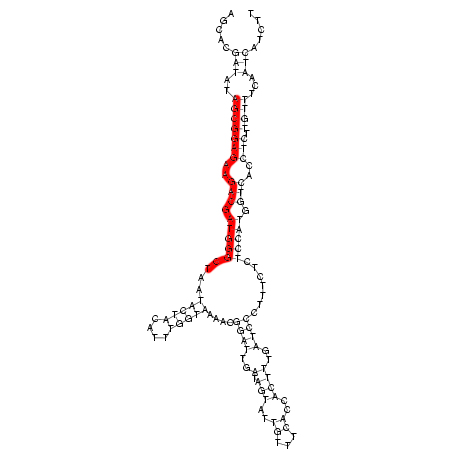


Secondary structure for ‘Z2’


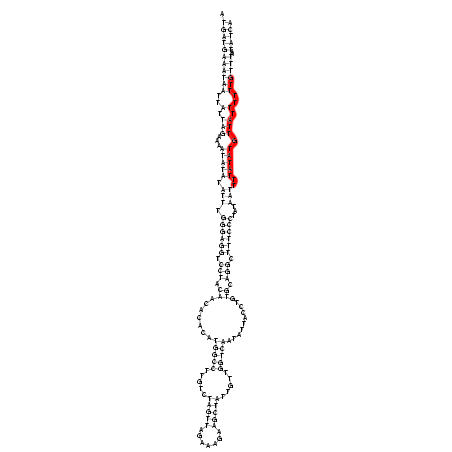


Secondary structure for ‘rgl-miR5138’


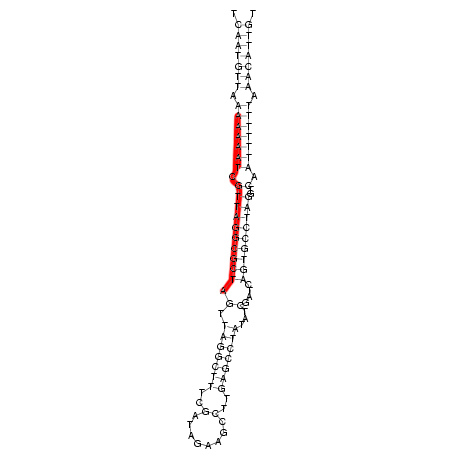


Secondary structure for ‘Z4’


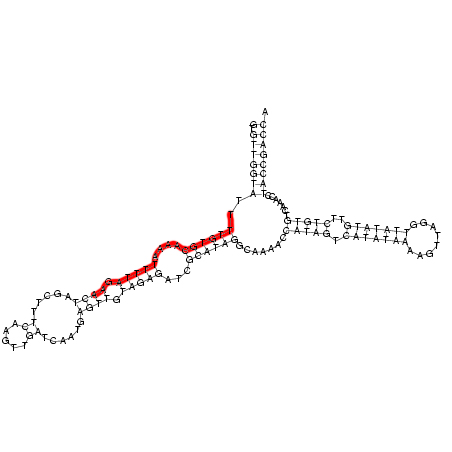


Secondary structure for ‘Z5’


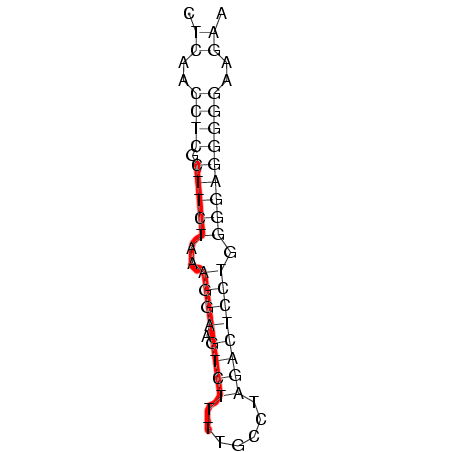


Secondary structure for ‘Z6’


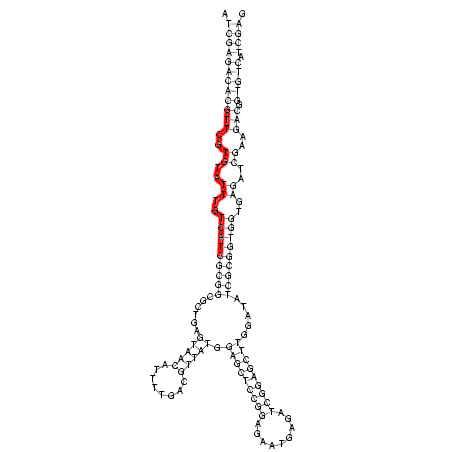


Secondary structure for ‘rgl-miR5139’


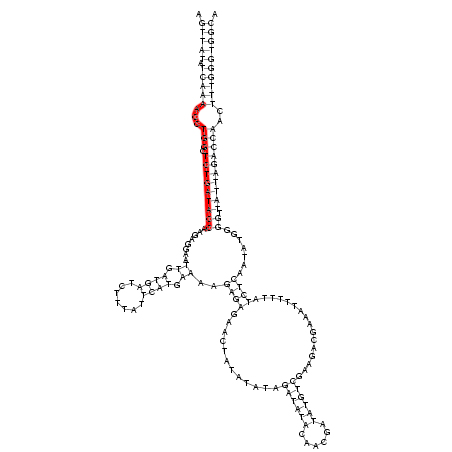


Secondary structure for ‘Z8’


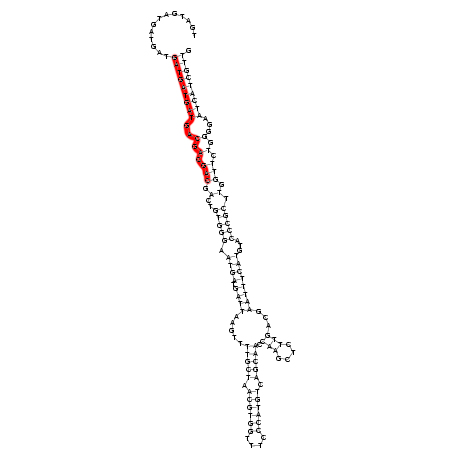


Secondary structure for ‘Z9’


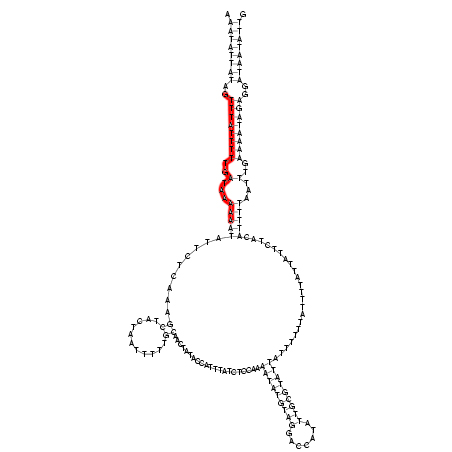


Secondary structure for ‘rgl-miR5140’


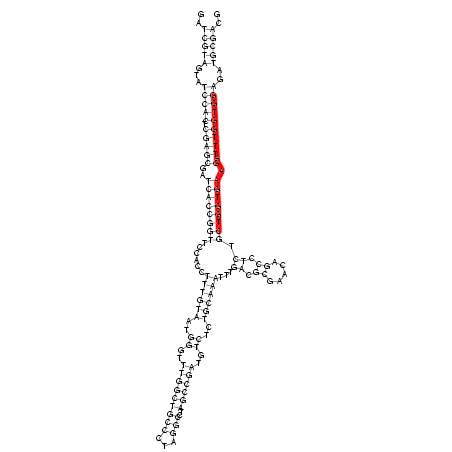


Secondary structure for ‘Z11’


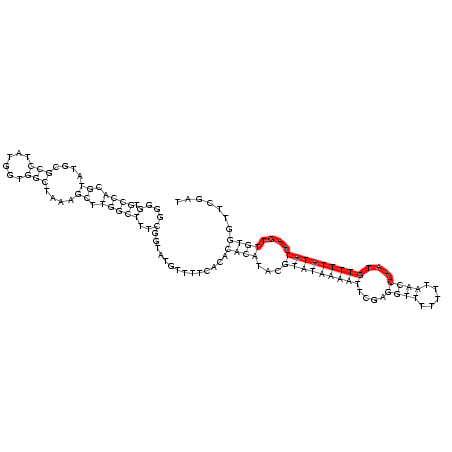


Secondary structure for ‘Z12’


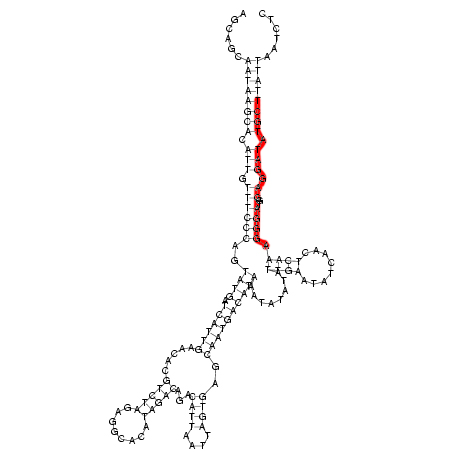


Secondary structure for ‘Z13’


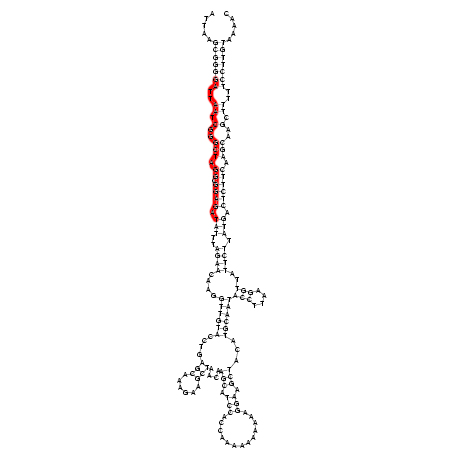


Secondary structure for ‘Z14’


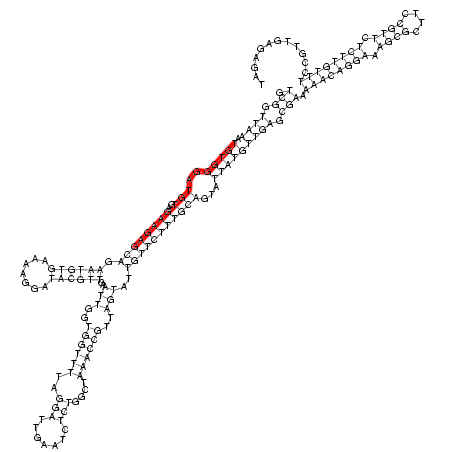


Sencondary sturcture for ‘rgl-miR5141’


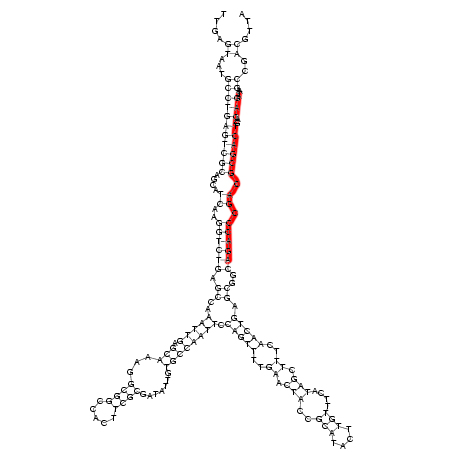


Secondary structure for ‘Z16’


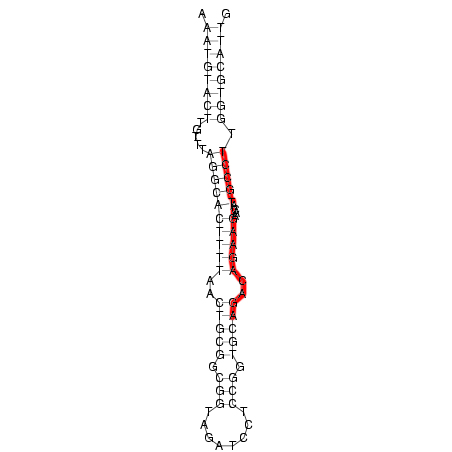


Secondary structure for ‘Z17’


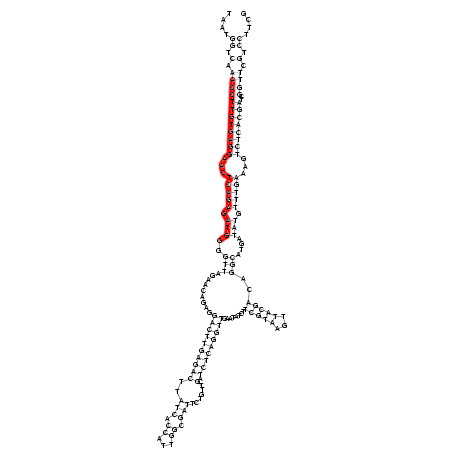


Secondary structure for ‘rgl-miR5142’


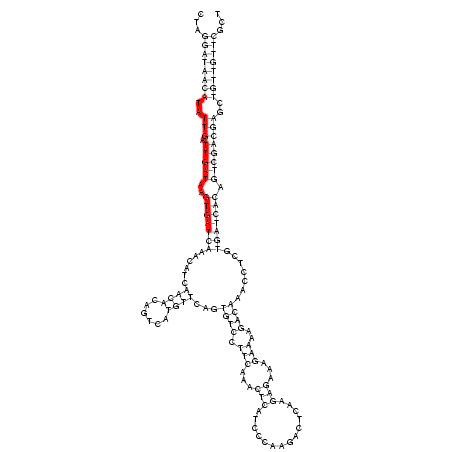

Supplement: Additional file 2 — Secondary structures of candidate miRNAs. [file 1471-2229-11-53-S2.DOC]
