# Supplementary material for: Differential miRNA expression in Rehmannia glutinosa plants subjected to continuous cropping
Source: BMC Plant Biol. 2011 Mar 26;11:53. doi: 10.1186/1471-2229-11-53 (PMC3078876; doi:10.1186/1471-2229-11-53)
Supplement: Additional file 3 — Potential target genes of 29 conserved miRNAs. [file 1471-2229-11-53-S3.DOC]

| **Additional file 3 - Potential target genes of 29 conserved miRNAs.** | | |
| --- | --- | --- |
| **22 potential target genes of miR156a** | | |
| sRNA_Acc. | Target_Acc. | Target_Desc. |
| submitted-miRNA | AT1G27360.1 | | Symbols: | squamosa promoter-binding protein-like 11 (SPL11) | chr1:9501958-9503856 FORWARD [PFAM] 688-918 PF03110.7 SBP domain; |
| submitted-miRNA | AT1G27360.2 | | Symbols: | squamosa promoter-binding protein-like 11 (SPL11) | chr1:9501064-9503856 FORWARD [PFAM] 648-878 PF03110.7 SBP domain; |
| submitted-miRNA | AT1G27360.3 | | Symbols: | squamosa promoter-binding protein-like 11 (SPL11) | chr1:9501764-9503856 FORWARD [PFAM] 675-905 PF03110.7 SBP domain; |
| submitted-miRNA | AT1G27360.4 | | Symbols: | squamosa promoter-binding protein-like 11 (SPL11) | chr1:9501808-9503856 FORWARD [PFAM] 735-965 PF03110.7 SBP domain; |
| submitted-miRNA | AT1G27370.1 | | Symbols: | squamosa promoter-binding protein-like 10 (SPL10) | chr1:9505188-9508267 REVERSE [PFAM] 1806-2036 PF03110.7 SBP domain; |
| submitted-miRNA | AT1G27370.2 | | Symbols: | squamosa promoter-binding protein-like 10 (SPL10) | chr1:9505189-9508468 REVERSE [PFAM] 884-1114 PF03110.7 SBP domain; |
| submitted-miRNA | AT1G27370.3 | | Symbols: | DNA binding | chr1:9505189-9507315 REVERSE [PFAM] 666-896 PF03110.7 SBP domain; |
| submitted-miRNA | AT1G27370.4 | | Symbols: | squamosa promoter-binding protein-like 10 (SPL10) | chr1:9505189-9508309 REVERSE [PFAM] 753-983 PF03110.7 SBP domain; |
| submitted-miRNA | AT1G69170.1 | | Symbols: | squamosa promoter-binding protein-like 6 (SPL6) | chr1:26008731-26010926 FORWARD [PFAM] 759-980 PF03110.7 SBP domain; |
| submitted-miRNA | AT2G42200.1 | | Symbols: | squamosa promoter-binding protein-like 9 (SPL9) | chr2:17594485-17596708 FORWARD [PFAM] 411-641 PF03110.7 SBP domain; |
| submitted-miRNA | AT3G57920.1 | | Symbols: | squamosa promoter-binding protein, putative | chr3:21455298-21457012 REVERSE [PFAM] 355-585 PF03110.7 SBP domain; |
| submitted-miRNA | AT5G43270.1 | | Symbols: SPL2 | SPL2 (SQUAMOSA PROMOTER BINDING PROTEIN-LIKE 2); DNA binding / transcription factor | chr5:17377529-17380201 REVERSE [PFAM] 681-902 PF03110.7 SBP domain; |
| submitted-miRNA | AT5G43270.2 | | Symbols: SPL2 | SPL2 (SQUAMOSA PROMOTER BINDING PROTEIN-LIKE 2); DNA binding / transcription factor | chr5:17377560-17381001 REVERSE [PFAM] 644-865 PF03110.7 SBP domain; |
| submitted-miRNA | AT5G43270.3 | | Symbols: SPL2 | SPL2 (SQUAMOSA PROMOTER BINDING PROTEIN-LIKE 2); DNA binding | chr5:17377560-17380191 REVERSE [PFAM] 587-808 PF03110.7 SBP domain; |
| submitted-miRNA | AT5G50570.1 | | Symbols: | squamosa promoter-binding protein, putative | chr5:20599309-20601785 REVERSE [PFAM] 525-758 PF03110.7 SBP domain; |
| submitted-miRNA | AT5G50570.2 | | Symbols: | squamosa promoter-binding protein, putative | chr5:20599309-20601106 REVERSE [PFAM] 390-623 PF03110.7 SBP domain; |
| submitted-miRNA | AT5G50670.1 | | Symbols: | squamosa promoter-binding protein, putative | chr5:20632930-20635065 REVERSE [PFAM] 463-696 PF03110.7 SBP domain; |
| submitted-miRNA | AT2G33810.1 | | Symbols: SPL3 | SPL3 (SQUAMOSA PROMOTER BINDING PROTEIN-LIKE 3); transcription factor | chr2:14312077-14313148 FORWARD [PFAM] 501-710 PF03110.7 SBP domain; |
| submitted-miRNA | AT3G28690.1 | | Symbols: | protein kinase, putative | chr3:10756276-10759105 FORWARD [PFAM] |
| submitted-miRNA | AT1G53160.1 | | Symbols: SPL4 | SPL4 (SQUAMOSA PROMOTER BINDING PROTEIN-LIKE 4); DNA binding / transcription factor | chr1:19810089-19810969 FORWARD [PFAM] 217-438 PF03110.7 SBP domain; |
| submitted-miRNA | AT5G11977.1 | | Symbols: MIR156E | MIR156E; miRNA | chr5:3867214-3867309 FORWARD [PFAM] |
| submitted-miRNA | AT5G10945.1 | | Symbols: MIR156D | MIR156D; miRNA | chr5:3456648-3456733 REVERSE [PFAM] |
| **21 potential target genes of miR156b** | | |
| sRNA_Acc. | Target_Acc. | Target_Desc. |
| submitted-miRNA | AT1G27360.1 | | Symbols: | squamosa promoter-binding protein-like 11 (SPL11) | chr1:9501958-9503856 FORWARD [PFAM] 688-918 PF03110.7 SBP domain; |
| submitted-miRNA | AT1G27360.2 | | Symbols: | squamosa promoter-binding protein-like 11 (SPL11) | chr1:9501064-9503856 FORWARD [PFAM] 648-878 PF03110.7 SBP domain; |
| submitted-miRNA | AT1G27360.3 | | Symbols: | squamosa promoter-binding protein-like 11 (SPL11) | chr1:9501764-9503856 FORWARD [PFAM] 675-905 PF03110.7 SBP domain; |
| submitted-miRNA | AT1G27360.4 | | Symbols: | squamosa promoter-binding protein-like 11 (SPL11) | chr1:9501808-9503856 FORWARD [PFAM] 735-965 PF03110.7 SBP domain; |
| submitted-miRNA | AT1G27370.1 | | Symbols: | squamosa promoter-binding protein-like 10 (SPL10) | chr1:9505188-9508267 REVERSE [PFAM] 1806-2036 PF03110.7 SBP domain; |
| submitted-miRNA | AT1G27370.2 | | Symbols: | squamosa promoter-binding protein-like 10 (SPL10) | chr1:9505189-9508468 REVERSE [PFAM] 884-1114 PF03110.7 SBP domain; |
| submitted-miRNA | AT1G27370.3 | | Symbols: | DNA binding | chr1:9505189-9507315 REVERSE [PFAM] 666-896 PF03110.7 SBP domain; |
| submitted-miRNA | AT1G27370.4 | | Symbols: | squamosa promoter-binding protein-like 10 (SPL10) | chr1:9505189-9508309 REVERSE [PFAM] 753-983 PF03110.7 SBP domain; |
| submitted-miRNA | AT1G69170.1 | | Symbols: | squamosa promoter-binding protein-like 6 (SPL6) | chr1:26008731-26010926 FORWARD [PFAM] 759-980 PF03110.7 SBP domain; |
| submitted-miRNA | AT2G42200.1 | | Symbols: | squamosa promoter-binding protein-like 9 (SPL9) | chr2:17594485-17596708 FORWARD [PFAM] 411-641 PF03110.7 SBP domain; |
| submitted-miRNA | AT3G57920.1 | | Symbols: | squamosa promoter-binding protein, putative | chr3:21455298-21457012 REVERSE [PFAM] 355-585 PF03110.7 SBP domain; |
| submitted-miRNA | AT5G43270.1 | | Symbols: SPL2 | SPL2 (SQUAMOSA PROMOTER BINDING PROTEIN-LIKE 2); DNA binding / transcription factor | chr5:17377529-17380201 REVERSE [PFAM] 681-902 PF03110.7 SBP domain; |
| submitted-miRNA | AT5G43270.2 | | Symbols: SPL2 | SPL2 (SQUAMOSA PROMOTER BINDING PROTEIN-LIKE 2); DNA binding / transcription factor | chr5:17377560-17381001 REVERSE [PFAM] 644-865 PF03110.7 SBP domain; |
| submitted-miRNA | AT5G43270.3 | | Symbols: SPL2 | SPL2 (SQUAMOSA PROMOTER BINDING PROTEIN-LIKE 2); DNA binding | chr5:17377560-17380191 REVERSE [PFAM] 587-808 PF03110.7 SBP domain; |
| submitted-miRNA | AT5G50570.1 | | Symbols: | squamosa promoter-binding protein, putative | chr5:20599309-20601785 REVERSE [PFAM] 525-758 PF03110.7 SBP domain; |
| submitted-miRNA | AT5G50570.2 | | Symbols: | squamosa promoter-binding protein, putative | chr5:20599309-20601106 REVERSE [PFAM] 390-623 PF03110.7 SBP domain; |
| submitted-miRNA | AT5G50670.1 | | Symbols: | squamosa promoter-binding protein, putative | chr5:20632930-20635065 REVERSE [PFAM] 463-696 PF03110.7 SBP domain; |
| submitted-miRNA | AT2G33810.1 | | Symbols: SPL3 | SPL3 (SQUAMOSA PROMOTER BINDING PROTEIN-LIKE 3); transcription factor | chr2:14312077-14313148 FORWARD [PFAM] 501-710 PF03110.7 SBP domain; |
| submitted-miRNA | AT3G28690.1 | | Symbols: | protein kinase, putative | chr3:10756276-10759105 FORWARD [PFAM] |
| submitted-miRNA | AT1G53160.1 | | Symbols: SPL4 | SPL4 (SQUAMOSA PROMOTER BINDING PROTEIN-LIKE 4); DNA binding / transcription factor | chr1:19810089-19810969 FORWARD [PFAM] 217-438 PF03110.7 SBP domain; |
| submitted-miRNA | AT5G11977.1 | | Symbols: MIR156E | MIR156E; miRNA | chr5:3867214-3867309 FORWARD [PFAM] |
| **22 potential target genes of miR156f** | | |
| sRNA_Acc. | Target_Acc. | Target_Desc. |
| submitted-miRNA | AT1G27360.1 | | Symbols: | squamosa promoter-binding protein-like 11 (SPL11) | chr1:9501958-9503856 FORWARD [PFAM] 688-918 PF03110.7 SBP domain; |
| submitted-miRNA | AT1G27360.2 | | Symbols: | squamosa promoter-binding protein-like 11 (SPL11) | chr1:9501064-9503856 FORWARD [PFAM] 648-878 PF03110.7 SBP domain; |
| submitted-miRNA | AT1G27360.3 | | Symbols: | squamosa promoter-binding protein-like 11 (SPL11) | chr1:9501764-9503856 FORWARD [PFAM] 675-905 PF03110.7 SBP domain; |
| submitted-miRNA | AT1G27360.4 | | Symbols: | squamosa promoter-binding protein-like 11 (SPL11) | chr1:9501808-9503856 FORWARD [PFAM] 735-965 PF03110.7 SBP domain; |
| submitted-miRNA | AT1G27370.1 | | Symbols: | squamosa promoter-binding protein-like 10 (SPL10) | chr1:9505188-9508267 REVERSE [PFAM] 1806-2036 PF03110.7 SBP domain; |
| submitted-miRNA | AT1G27370.2 | | Symbols: | squamosa promoter-binding protein-like 10 (SPL10) | chr1:9505189-9508468 REVERSE [PFAM] 884-1114 PF03110.7 SBP domain; |
| submitted-miRNA | AT1G27370.3 | | Symbols: | DNA binding | chr1:9505189-9507315 REVERSE [PFAM] 666-896 PF03110.7 SBP domain; |
| submitted-miRNA | AT1G27370.4 | | Symbols: | squamosa promoter-binding protein-like 10 (SPL10) | chr1:9505189-9508309 REVERSE [PFAM] 753-983 PF03110.7 SBP domain; |
| submitted-miRNA | AT2G33810.1 | | Symbols: SPL3 | SPL3 (SQUAMOSA PROMOTER BINDING PROTEIN-LIKE 3); transcription factor | chr2:14312077-14313148 FORWARD [PFAM] 501-710 PF03110.7 SBP domain; |
| submitted-miRNA | AT2G42200.1 | | Symbols: | squamosa promoter-binding protein-like 9 (SPL9) | chr2:17594485-17596708 FORWARD [PFAM] 411-641 PF03110.7 SBP domain; |
| submitted-miRNA | AT3G57920.1 | | Symbols: | squamosa promoter-binding protein, putative | chr3:21455298-21457012 REVERSE [PFAM] 355-585 PF03110.7 SBP domain; |
| submitted-miRNA | AT5G43270.1 | | Symbols: SPL2 | SPL2 (SQUAMOSA PROMOTER BINDING PROTEIN-LIKE 2); DNA binding / transcription factor | chr5:17377529-17380201 REVERSE [PFAM] 681-902 PF03110.7 SBP domain; |
| submitted-miRNA | AT5G43270.2 | | Symbols: SPL2 | SPL2 (SQUAMOSA PROMOTER BINDING PROTEIN-LIKE 2); DNA binding / transcription factor | chr5:17377560-17381001 REVERSE [PFAM] 644-865 PF03110.7 SBP domain; |
| submitted-miRNA | AT5G43270.3 | | Symbols: SPL2 | SPL2 (SQUAMOSA PROMOTER BINDING PROTEIN-LIKE 2); DNA binding | chr5:17377560-17380191 REVERSE [PFAM] 587-808 PF03110.7 SBP domain; |
| submitted-miRNA | AT1G69170.1 | | Symbols: | squamosa promoter-binding protein-like 6 (SPL6) | chr1:26008731-26010926 FORWARD [PFAM] 759-980 PF03110.7 SBP domain; |
| submitted-miRNA | AT1G53160.1 | | Symbols: SPL4 | SPL4 (SQUAMOSA PROMOTER BINDING PROTEIN-LIKE 4); DNA binding / transcription factor | chr1:19810089-19810969 FORWARD [PFAM] 217-438 PF03110.7 SBP domain; |
| submitted-miRNA | AT5G50570.1 | | Symbols: | squamosa promoter-binding protein, putative | chr5:20599309-20601785 REVERSE [PFAM] 525-758 PF03110.7 SBP domain; |
| submitted-miRNA | AT5G50570.2 | | Symbols: | squamosa promoter-binding protein, putative | chr5:20599309-20601106 REVERSE [PFAM] 390-623 PF03110.7 SBP domain; |
| submitted-miRNA | AT5G50670.1 | | Symbols: | squamosa promoter-binding protein, putative | chr5:20632930-20635065 REVERSE [PFAM] 463-696 PF03110.7 SBP domain; |
| submitted-miRNA | AT3G17330.1 | | Symbols: ECT6 | ECT6 (evolutionarily conserved C-terminal region 6) | chr3:5916946-5920194 REVERSE [PFAM] |
| submitted-miRNA | AT3G17330.2 | | Symbols: ECT6 | ECT6 (evolutionarily conserved C-terminal region 6) | chr3:5916946-5920194 REVERSE [PFAM] |
| submitted-miRNA | AT3G28690.1 | | Symbols: | protein kinase, putative | chr3:10756276-10759105 FORWARD [PFAM] |
| **19 potential target genes of miR157a** | | |
| sRNA_Acc. | Target_Acc. | Target_Desc. |
| submitted-miRNA | AT1G27360.1 | | Symbols: | squamosa promoter-binding protein-like 11 (SPL11) | chr1:9501958-9503856 FORWARD [PFAM] 688-918 PF03110.7 SBP domain; |
| submitted-miRNA | AT1G27360.2 | | Symbols: | squamosa promoter-binding protein-like 11 (SPL11) | chr1:9501064-9503856 FORWARD [PFAM] 648-878 PF03110.7 SBP domain; |
| submitted-miRNA | AT1G27360.3 | | Symbols: | squamosa promoter-binding protein-like 11 (SPL11) | chr1:9501764-9503856 FORWARD [PFAM] 675-905 PF03110.7 SBP domain; |
| submitted-miRNA | AT1G27360.4 | | Symbols: | squamosa promoter-binding protein-like 11 (SPL11) | chr1:9501808-9503856 FORWARD [PFAM] 735-965 PF03110.7 SBP domain; |
| submitted-miRNA | AT1G27370.1 | | Symbols: | squamosa promoter-binding protein-like 10 (SPL10) | chr1:9505188-9508267 REVERSE [PFAM] 1806-2036 PF03110.7 SBP domain; |
| submitted-miRNA | AT1G27370.2 | | Symbols: | squamosa promoter-binding protein-like 10 (SPL10) | chr1:9505189-9508468 REVERSE [PFAM] 884-1114 PF03110.7 SBP domain; |
| submitted-miRNA | AT1G27370.3 | | Symbols: | DNA binding | chr1:9505189-9507315 REVERSE [PFAM] 666-896 PF03110.7 SBP domain; |
| submitted-miRNA | AT1G27370.4 | | Symbols: | squamosa promoter-binding protein-like 10 (SPL10) | chr1:9505189-9508309 REVERSE [PFAM] 753-983 PF03110.7 SBP domain; |
| submitted-miRNA | AT2G42200.1 | | Symbols: | squamosa promoter-binding protein-like 9 (SPL9) | chr2:17594485-17596708 FORWARD [PFAM] 411-641 PF03110.7 SBP domain; |
| submitted-miRNA | AT3G57920.1 | | Symbols: | squamosa promoter-binding protein, putative | chr3:21455298-21457012 REVERSE [PFAM] 355-585 PF03110.7 SBP domain; |
| submitted-miRNA | AT5G43270.1 | | Symbols: SPL2 | SPL2 (SQUAMOSA PROMOTER BINDING PROTEIN-LIKE 2); DNA binding / transcription factor | chr5:17377529-17380201 REVERSE [PFAM] 681-902 PF03110.7 SBP domain; |
| submitted-miRNA | AT5G43270.2 | | Symbols: SPL2 | SPL2 (SQUAMOSA PROMOTER BINDING PROTEIN-LIKE 2); DNA binding / transcription factor | chr5:17377560-17381001 REVERSE [PFAM] 644-865 PF03110.7 SBP domain; |
| submitted-miRNA | AT5G43270.3 | | Symbols: SPL2 | SPL2 (SQUAMOSA PROMOTER BINDING PROTEIN-LIKE 2); DNA binding | chr5:17377560-17380191 REVERSE [PFAM] 587-808 PF03110.7 SBP domain; |
| submitted-miRNA | AT1G69170.1 | | Symbols: | squamosa promoter-binding protein-like 6 (SPL6) | chr1:26008731-26010926 FORWARD [PFAM] 759-980 PF03110.7 SBP domain; |
| submitted-miRNA | AT1G53160.1 | | Symbols: SPL4 | SPL4 (SQUAMOSA PROMOTER BINDING PROTEIN-LIKE 4); DNA binding / transcription factor | chr1:19810089-19810969 FORWARD [PFAM] 217-438 PF03110.7 SBP domain; |
| submitted-miRNA | AT5G08620.1 | | Symbols: | DEAD box RNA helicase (RH25) | chr5:2794458-2797661 FORWARD [PFAM] |
| submitted-miRNA | AT5G50570.1 | | Symbols: | squamosa promoter-binding protein, putative | chr5:20599309-20601785 REVERSE [PFAM] 525-758 PF03110.7 SBP domain; |
| submitted-miRNA | AT5G50570.2 | | Symbols: | squamosa promoter-binding protein, putative | chr5:20599309-20601106 REVERSE [PFAM] 390-623 PF03110.7 SBP domain; |
| submitted-miRNA | AT5G50670.1 | | Symbols: | squamosa promoter-binding protein, putative | chr5:20632930-20635065 REVERSE [PFAM] 463-696 PF03110.7 SBP domain; |
| **21 potential target genes of miR157b** | | |
| sRNA_Acc. | Target_Acc. | Target_Desc. |
| submitted-miRNA | AT1G27360.1 | | Symbols: | squamosa promoter-binding protein-like 11 (SPL11) | chr1:9501958-9503856 FORWARD [PFAM] 688-918 PF03110.7 SBP domain; |
| submitted-miRNA | AT1G27360.2 | | Symbols: | squamosa promoter-binding protein-like 11 (SPL11) | chr1:9501064-9503856 FORWARD [PFAM] 648-878 PF03110.7 SBP domain; |
| submitted-miRNA | AT1G27360.3 | | Symbols: | squamosa promoter-binding protein-like 11 (SPL11) | chr1:9501764-9503856 FORWARD [PFAM] 675-905 PF03110.7 SBP domain; |
| submitted-miRNA | AT1G27360.4 | | Symbols: | squamosa promoter-binding protein-like 11 (SPL11) | chr1:9501808-9503856 FORWARD [PFAM] 735-965 PF03110.7 SBP domain; |
| submitted-miRNA | AT1G27370.1 | | Symbols: | squamosa promoter-binding protein-like 10 (SPL10) | chr1:9505188-9508267 REVERSE [PFAM] 1806-2036 PF03110.7 SBP domain; |
| submitted-miRNA | AT1G27370.2 | | Symbols: | squamosa promoter-binding protein-like 10 (SPL10) | chr1:9505189-9508468 REVERSE [PFAM] 884-1114 PF03110.7 SBP domain; |
| submitted-miRNA | AT1G27370.3 | | Symbols: | DNA binding | chr1:9505189-9507315 REVERSE [PFAM] 666-896 PF03110.7 SBP domain; |
| submitted-miRNA | AT1G27370.4 | | Symbols: | squamosa promoter-binding protein-like 10 (SPL10) | chr1:9505189-9508309 REVERSE [PFAM] 753-983 PF03110.7 SBP domain; |
| submitted-miRNA | AT1G69170.1 | | Symbols: | squamosa promoter-binding protein-like 6 (SPL6) | chr1:26008731-26010926 FORWARD [PFAM] 759-980 PF03110.7 SBP domain; |
| submitted-miRNA | AT2G42200.1 | | Symbols: | squamosa promoter-binding protein-like 9 (SPL9) | chr2:17594485-17596708 FORWARD [PFAM] 411-641 PF03110.7 SBP domain; |
| submitted-miRNA | AT3G57920.1 | | Symbols: | squamosa promoter-binding protein, putative | chr3:21455298-21457012 REVERSE [PFAM] 355-585 PF03110.7 SBP domain; |
| submitted-miRNA | AT5G43270.1 | | Symbols: SPL2 | SPL2 (SQUAMOSA PROMOTER BINDING PROTEIN-LIKE 2); DNA binding / transcription factor | chr5:17377529-17380201 REVERSE [PFAM] 681-902 PF03110.7 SBP domain; |
| submitted-miRNA | AT5G43270.2 | | Symbols: SPL2 | SPL2 (SQUAMOSA PROMOTER BINDING PROTEIN-LIKE 2); DNA binding / transcription factor | chr5:17377560-17381001 REVERSE [PFAM] 644-865 PF03110.7 SBP domain; |
| submitted-miRNA | AT5G43270.3 | | Symbols: SPL2 | SPL2 (SQUAMOSA PROMOTER BINDING PROTEIN-LIKE 2); DNA binding | chr5:17377560-17380191 REVERSE [PFAM] 587-808 PF03110.7 SBP domain; |
| submitted-miRNA | AT5G50570.1 | | Symbols: | squamosa promoter-binding protein, putative | chr5:20599309-20601785 REVERSE [PFAM] 525-758 PF03110.7 SBP domain; |
| submitted-miRNA | AT5G50570.2 | | Symbols: | squamosa promoter-binding protein, putative | chr5:20599309-20601106 REVERSE [PFAM] 390-623 PF03110.7 SBP domain; |
| submitted-miRNA | AT5G50670.1 | | Symbols: | squamosa promoter-binding protein, putative | chr5:20632930-20635065 REVERSE [PFAM] 463-696 PF03110.7 SBP domain; |
| submitted-miRNA | AT1G53160.1 | | Symbols: SPL4 | SPL4 (SQUAMOSA PROMOTER BINDING PROTEIN-LIKE 4); DNA binding / transcription factor | chr1:19810089-19810969 FORWARD [PFAM] 217-438 PF03110.7 SBP domain; |
| submitted-miRNA | AT3G18217.1 | | Symbols: MIR157c | MIR157c; miRNA | chr3:6244535-6244699 REVERSE [PFAM] |
| submitted-miRNA | AT5G08620.1 | | Symbols: | DEAD box RNA helicase (RH25) | chr5:2794458-2797661 FORWARD [PFAM] |
| submitted-miRNA | AT2G03750.1 | | Symbols: | sulfotransferase family protein | chr2:1147804-1149058 REVERSE [PFAM] |
| **19 potential target genes of miR157c** | | |
| sRNA_Acc. | Target_Acc. | Target_Desc. |
| submitted-miRNA | AT1G27360.1 | | Symbols: | squamosa promoter-binding protein-like 11 (SPL11) | chr1:9501958-9503856 FORWARD [PFAM] 688-918 PF03110.7 SBP domain; |
| submitted-miRNA | AT1G27360.2 | | Symbols: | squamosa promoter-binding protein-like 11 (SPL11) | chr1:9501064-9503856 FORWARD [PFAM] 648-878 PF03110.7 SBP domain; |
| submitted-miRNA | AT1G27360.3 | | Symbols: | squamosa promoter-binding protein-like 11 (SPL11) | chr1:9501764-9503856 FORWARD [PFAM] 675-905 PF03110.7 SBP domain; |
| submitted-miRNA | AT1G27360.4 | | Symbols: | squamosa promoter-binding protein-like 11 (SPL11) | chr1:9501808-9503856 FORWARD [PFAM] 735-965 PF03110.7 SBP domain; |
| submitted-miRNA | AT1G27370.1 | | Symbols: | squamosa promoter-binding protein-like 10 (SPL10) | chr1:9505188-9508267 REVERSE [PFAM] 1806-2036 PF03110.7 SBP domain; |
| submitted-miRNA | AT1G27370.2 | | Symbols: | squamosa promoter-binding protein-like 10 (SPL10) | chr1:9505189-9508468 REVERSE [PFAM] 884-1114 PF03110.7 SBP domain; |
| submitted-miRNA | AT1G27370.3 | | Symbols: | DNA binding | chr1:9505189-9507315 REVERSE [PFAM] 666-896 PF03110.7 SBP domain; |
| submitted-miRNA | AT1G27370.4 | | Symbols: | squamosa promoter-binding protein-like 10 (SPL10) | chr1:9505189-9508309 REVERSE [PFAM] 753-983 PF03110.7 SBP domain; |
| submitted-miRNA | AT2G42200.1 | | Symbols: | squamosa promoter-binding protein-like 9 (SPL9) | chr2:17594485-17596708 FORWARD [PFAM] 411-641 PF03110.7 SBP domain; |
| submitted-miRNA | AT3G57920.1 | | Symbols: | squamosa promoter-binding protein, putative | chr3:21455298-21457012 REVERSE [PFAM] 355-585 PF03110.7 SBP domain; |
| submitted-miRNA | AT5G43270.1 | | Symbols: SPL2 | SPL2 (SQUAMOSA PROMOTER BINDING PROTEIN-LIKE 2); DNA binding / transcription factor | chr5:17377529-17380201 REVERSE [PFAM] 681-902 PF03110.7 SBP domain; |
| submitted-miRNA | AT5G43270.2 | | Symbols: SPL2 | SPL2 (SQUAMOSA PROMOTER BINDING PROTEIN-LIKE 2); DNA binding / transcription factor | chr5:17377560-17381001 REVERSE [PFAM] 644-865 PF03110.7 SBP domain; |
| submitted-miRNA | AT5G43270.3 | | Symbols: SPL2 | SPL2 (SQUAMOSA PROMOTER BINDING PROTEIN-LIKE 2); DNA binding | chr5:17377560-17380191 REVERSE [PFAM] 587-808 PF03110.7 SBP domain; |
| submitted-miRNA | AT1G69170.1 | | Symbols: | squamosa promoter-binding protein-like 6 (SPL6) | chr1:26008731-26010926 FORWARD [PFAM] 759-980 PF03110.7 SBP domain; |
| submitted-miRNA | AT1G53160.1 | | Symbols: SPL4 | SPL4 (SQUAMOSA PROMOTER BINDING PROTEIN-LIKE 4); DNA binding / transcription factor | chr1:19810089-19810969 FORWARD [PFAM] 217-438 PF03110.7 SBP domain; |
| submitted-miRNA | AT5G08620.1 | | Symbols: | DEAD box RNA helicase (RH25) | chr5:2794458-2797661 FORWARD [PFAM] |
| submitted-miRNA | AT5G50570.1 | | Symbols: | squamosa promoter-binding protein, putative | chr5:20599309-20601785 REVERSE [PFAM] 525-758 PF03110.7 SBP domain; |
| submitted-miRNA | AT5G50570.2 | | Symbols: | squamosa promoter-binding protein, putative | chr5:20599309-20601106 REVERSE [PFAM] 390-623 PF03110.7 SBP domain; |
| submitted-miRNA | AT5G50670.1 | | Symbols: | squamosa promoter-binding protein, putative | chr5:20632930-20635065 REVERSE [PFAM] 463-696 PF03110.7 SBP domain; |
| **22 potential target genes of miR157d** | | |
| sRNA_Acc. | Target_Acc. | Target_Desc. |
| submitted-miRNA | AT1G27360.1 | | Symbols: | squamosa promoter-binding protein-like 11 (SPL11) | chr1:9501958-9503856 FORWARD [PFAM] 688-918 PF03110.7 SBP domain; |
| submitted-miRNA | AT1G27360.2 | | Symbols: | squamosa promoter-binding protein-like 11 (SPL11) | chr1:9501064-9503856 FORWARD [PFAM] 648-878 PF03110.7 SBP domain; |
| submitted-miRNA | AT1G27360.3 | | Symbols: | squamosa promoter-binding protein-like 11 (SPL11) | chr1:9501764-9503856 FORWARD [PFAM] 675-905 PF03110.7 SBP domain; |
| submitted-miRNA | AT1G27360.4 | | Symbols: | squamosa promoter-binding protein-like 11 (SPL11) | chr1:9501808-9503856 FORWARD [PFAM] 735-965 PF03110.7 SBP domain; |
| submitted-miRNA | AT1G27370.1 | | Symbols: | squamosa promoter-binding protein-like 10 (SPL10) | chr1:9505188-9508267 REVERSE [PFAM] 1806-2036 PF03110.7 SBP domain; |
| submitted-miRNA | AT1G27370.2 | | Symbols: | squamosa promoter-binding protein-like 10 (SPL10) | chr1:9505189-9508468 REVERSE [PFAM] 884-1114 PF03110.7 SBP domain; |
| submitted-miRNA | AT1G27370.3 | | Symbols: | DNA binding | chr1:9505189-9507315 REVERSE [PFAM] 666-896 PF03110.7 SBP domain; |
| submitted-miRNA | AT1G27370.4 | | Symbols: | squamosa promoter-binding protein-like 10 (SPL10) | chr1:9505189-9508309 REVERSE [PFAM] 753-983 PF03110.7 SBP domain; |
| submitted-miRNA | AT1G69170.1 | | Symbols: | squamosa promoter-binding protein-like 6 (SPL6) | chr1:26008731-26010926 FORWARD [PFAM] 759-980 PF03110.7 SBP domain; |
| submitted-miRNA | AT2G42200.1 | | Symbols: | squamosa promoter-binding protein-like 9 (SPL9) | chr2:17594485-17596708 FORWARD [PFAM] 411-641 PF03110.7 SBP domain; |
| submitted-miRNA | AT3G57920.1 | | Symbols: | squamosa promoter-binding protein, putative | chr3:21455298-21457012 REVERSE [PFAM] 355-585 PF03110.7 SBP domain; |
| submitted-miRNA | AT5G43270.1 | | Symbols: SPL2 | SPL2 (SQUAMOSA PROMOTER BINDING PROTEIN-LIKE 2); DNA binding / transcription factor | chr5:17377529-17380201 REVERSE [PFAM] 681-902 PF03110.7 SBP domain; |
| submitted-miRNA | AT5G43270.2 | | Symbols: SPL2 | SPL2 (SQUAMOSA PROMOTER BINDING PROTEIN-LIKE 2); DNA binding / transcription factor | chr5:17377560-17381001 REVERSE [PFAM] 644-865 PF03110.7 SBP domain; |
| submitted-miRNA | AT5G43270.3 | | Symbols: SPL2 | SPL2 (SQUAMOSA PROMOTER BINDING PROTEIN-LIKE 2); DNA binding | chr5:17377560-17380191 REVERSE [PFAM] 587-808 PF03110.7 SBP domain; |
| submitted-miRNA | AT5G50570.1 | | Symbols: | squamosa promoter-binding protein, putative | chr5:20599309-20601785 REVERSE [PFAM] 525-758 PF03110.7 SBP domain; |
| submitted-miRNA | AT5G50570.2 | | Symbols: | squamosa promoter-binding protein, putative | chr5:20599309-20601106 REVERSE [PFAM] 390-623 PF03110.7 SBP domain; |
| submitted-miRNA | AT5G50670.1 | | Symbols: | squamosa promoter-binding protein, putative | chr5:20632930-20635065 REVERSE [PFAM] 463-696 PF03110.7 SBP domain; |
| submitted-miRNA | AT1G48742.1 | | Symbols: MIR157d | MIR157d; miRNA | chr1:18030506-18030677 REVERSE [PFAM] |
| submitted-miRNA | AT1G53160.1 | | Symbols: SPL4 | SPL4 (SQUAMOSA PROMOTER BINDING PROTEIN-LIKE 4); DNA binding / transcription factor | chr1:19810089-19810969 FORWARD [PFAM] 217-438 PF03110.7 SBP domain; |
| submitted-miRNA | AT3G18217.1 | | Symbols: MIR157c | MIR157c; miRNA | chr3:6244535-6244699 REVERSE [PFAM] |
| submitted-miRNA | AT5G08620.1 | | Symbols: | DEAD box RNA helicase (RH25) | chr5:2794458-2797661 FORWARD [PFAM] |
| submitted-miRNA | AT2G03750.1 | | Symbols: | sulfotransferase family protein | chr2:1147804-1149058 REVERSE [PFAM] |
| **3 potential target genes of miR160a** | | |
| RNA_Acc. | Target_Acc. | Target_Desc. |
| submitted-miRNA | AT1G77850.1 | | Symbols: ARF17 | ARF17 (AUXIN RESPONSE FACTOR 17); transcription factor | chr1:29277207-29280313 FORWARD [PFAM] |
| submitted-miRNA | AT2G28350.1 | | Symbols: ARF10 | ARF10 (AUXIN RESPONSE FACTOR 10); miRNA binding / transcription factor | chr2:12121408-12123925 FORWARD [PFAM] |
| submitted-miRNA | AT4G30080.1 | | Symbols: ARF16 | ARF16 (AUXIN RESPONSE FACTOR 16); miRNA binding / transcription factor | chr4:14703207-14706342 REVERSE [PFAM] |
| **3 potential target genes of miR160b** | | |
| sRNA_Acc. | Target_Acc. | Target_Desc. |
| submitted-miRNA | AT1G77850.1 | | Symbols: ARF17 | ARF17 (AUXIN RESPONSE FACTOR 17); transcription factor | chr1:29277207-29280313 FORWARD [PFAM] |
| submitted-miRNA | AT4G30080.1 | | Symbols: ARF16 | ARF16 (AUXIN RESPONSE FACTOR 16); miRNA binding / transcription factor | chr4:14703207-14706342 REVERSE [PFAM] |
| submitted-miRNA | AT2G28350.1 | | Symbols: ARF10 | ARF10 (AUXIN RESPONSE FACTOR 10); miRNA binding / transcription factor | chr2:12121408-12123925 FORWARD [PFAM] |
| **3 potential target genes of miR160c** | | |
| sRNA_Acc. | Target_Acc. | Target_Desc. |
| submitted-miRNA | AT1G77850.1 | | Symbols: ARF17 | ARF17 (AUXIN RESPONSE FACTOR 17); transcription factor | chr1:29277207-29280313 FORWARD [PFAM] |
| submitted-miRNA | AT2G28350.1 | | Symbols: ARF10 | ARF10 (AUXIN RESPONSE FACTOR 10); miRNA binding / transcription factor | chr2:12121408-12123925 FORWARD [PFAM] |
| submitted-miRNA | AT4G30080.1 | | Symbols: ARF16 | ARF16 (AUXIN RESPONSE FACTOR 16); miRNA binding / transcription factor | chr4:14703207-14706342 REVERSE [PFAM] |
| **11 potential target genes of miR164b** | | |
| sRNA_Acc. | Target_Acc. | Target_Desc. |
| submitted-miRNA | AT1G56010.1 | | Symbols: ANAC021, ANAC022, NAC1 | NAC1 (Arabidopsis NAC domain containing protein 21, Arabidopsis NAC domain containing protein 22); transcription factor | chr1:20950236-20951705 REVERSE [PFAM] 332-550 PF02365.8 No apical meristem (NAM) protein; |
| submitted-miRNA | AT1G56010.2 | | Symbols: ANAC021, ANAC022, NAC1 | NAC1 (Arabidopsis NAC domain containing protein 21, Arabidopsis NAC domain containing protein 22); transcription factor | chr1:20950236-20952946 REVERSE [PFAM] 192-575 PF02365.8 No apical meristem (NAM) protein; |
| submitted-miRNA | AT3G15170.1 | | Symbols: ANAC054, ATNAC1, CUC1 | CUC1 (CUP-SHAPED COTYLEDON1); transcription factor | chr3:5109890-5111454 FORWARD [PFAM] 87-473 PF02365.8 No apical meristem (NAM) protein; |
| submitted-miRNA | AT5G53950.1 | | Symbols: ANAC098, CUC2 | CUC2 (CUP-SHAPED COTYLEDON 2); transcription factor | chr5:21919192-21921049 REVERSE [PFAM] 77-463 PF02365.8 No apical meristem (NAM) protein; |
| submitted-miRNA | AT3G12977.1 | | Symbols: | DNA binding | chr3:4143836-4145974 FORWARD [PFAM] 55-435 PF02365.8 No apical meristem (NAM) protein; |
| submitted-miRNA | AT5G07680.1 | | Symbols: ANAC079, ATNAC4, ANAC080 | ANAC079/ANAC080/ATNAC4 (Arabidopsis NAC domain containing protein 79, Arabidopsis NAC domain containing protein 80); transcription factor | chr5:2435876-2437481 FORWARD [PFAM] 266-646 PF02365.8 No apical meristem (NAM) protein; |
| submitted-miRNA | AT5G07680.2 | | Symbols: ANAC079, ATNAC4, ANAC080 | ANAC079/ANAC080/ATNAC4 (Arabidopsis NAC domain containing protein 79, Arabidopsis NAC domain containing protein 80); transcription factor | chr5:2435984-2437500 FORWARD [PFAM] 74-454 PF02365.8 No apical meristem (NAM) protein; |
| submitted-miRNA | AT5G61430.1 | | Symbols: ANAC100, ATNAC5 | ANAC100/ATNAC5 (Arabidopsis NAC domain containing protein 100); transcription factor | chr5:24718348-24719987 REVERSE [PFAM] 254-634 PF02365.8 No apical meristem (NAM) protein; |
| submitted-miRNA | AT5G39610.1 | | Symbols: ANAC092, ATNAC6, ATNAC2 | ANAC092/ATNAC2/ATNAC6 (Arabidopsis NAC domain containing protein 92); protein heterodimerization/ protein homodimerization/ transcription factor | chr5:15875628-15877016 REVERSE [PFAM] 196-576 PF02365.8 No apical meristem (NAM) protein; |
| submitted-miRNA | AT1G10530.1 | | Symbols: | similar to unknown protein [Arabidopsis thaliana] (TAIR:AT1G60010.1); similar to T10O24.15, related [Lycopersicon esculentum] (GB:AAX95759.1) | chr1:3471457-3472673 REVERSE [PFAM] |
| submitted-miRNA | AT2G37960.1 | | Symbols: | similar to unknown protein [Arabidopsis thaliana] (TAIR:AT3G54060.2); similar to conserved hypothetical protein [Medicago truncatula] (GB:ABE83850.1); contains domain Cullin repeat (SSF74788) | chr2:15893626-15896298 REVERSE [PFAM] |
| **7 potential target genes of miR166a** | | |
| sRNA_Acc. | Target_Acc. | Target_Desc. |
| submitted-miRNA | AT1G52150.1 | | Symbols: ATHB15, CNA, ICU4, ATHB-15 | ATHB-15 (INCURVATA 4); DNA binding / transcription factor | chr1:19413380-19418347 REVERSE [PFAM] 2782-3225 PF08670.4 MEKHLA domain; |
| submitted-miRNA | AT1G52150.2 | | Symbols: ATHB15, CNA, ICU4, ATHB-15 | ATHB-15 (INCURVATA 4); DNA binding / transcription factor | chr1:19413380-19418347 REVERSE [PFAM] 2785-3228 PF08670.4 MEKHLA domain; |
| submitted-miRNA | AT1G52150.3 | | Symbols: ATHB15, CNA, ICU4, ATHB-15 | ATHB-15 (INCURVATA 4) | chr1:19413380-19418347 REVERSE [PFAM] 2782-3081 PF08670.4 MEKHLA domain; |
| submitted-miRNA | AT1G30490.1 | | Symbols: ATHB9 | PHV (PHAVOLUTA); DNA binding / transcription factor | chr1:10796117-10800943 REVERSE [PFAM] |
| submitted-miRNA | AT2G34710.1 | | Symbols: ATHB14, ATHB-14, PHB-1D, PHB | PHB (PHABULOSA); DNA binding / transcription factor | chr2:14646402-14651352 REVERSE [PFAM] |
| submitted-miRNA | AT4G32880.1 | | Symbols: ATHB8, ATHB-8 | ATHB-8 (HOMEOBOX GENE 8); DNA binding / transcription factor | chr4:15863456-15868629 REVERSE [PFAM] |
| submitted-miRNA | AT5G60690.1 | | Symbols: IFL, IFL1, REV | REV (REVOLUTA); DNA binding / lipid binding / transcription factor | chr5:24414249-24419421 FORWARD [PFAM] |
| **11 potential target genes of miR166b** | | |
| sRNA_Acc. | Target_Acc. | Target_Desc. |
| submitted-miRNA | AT1G52150.1 | | Symbols: ATHB15, CNA, ICU4, ATHB-15 | ATHB-15 (INCURVATA 4); DNA binding / transcription factor | chr1:19413380-19418347 REVERSE [PFAM] 2782-3225 PF08670.4 MEKHLA domain; |
| submitted-miRNA | AT1G52150.2 | | Symbols: ATHB15, CNA, ICU4, ATHB-15 | ATHB-15 (INCURVATA 4); DNA binding / transcription factor | chr1:19413380-19418347 REVERSE [PFAM] 2785-3228 PF08670.4 MEKHLA domain; |
| submitted-miRNA | AT1G52150.3 | | Symbols: ATHB15, CNA, ICU4, ATHB-15 | ATHB-15 (INCURVATA 4) | chr1:19413380-19418347 REVERSE [PFAM] 2782-3081 PF08670.4 MEKHLA domain; |
| submitted-miRNA | AT1G30490.1 | | Symbols: ATHB9 | PHV (PHAVOLUTA); DNA binding / transcription factor | chr1:10796117-10800943 REVERSE [PFAM] |
| submitted-miRNA | AT2G34710.1 | | Symbols: ATHB14, ATHB-14, PHB-1D, PHB | PHB (PHABULOSA); DNA binding / transcription factor | chr2:14646402-14651352 REVERSE [PFAM] |
| submitted-miRNA | AT4G32880.1 | | Symbols: ATHB8, ATHB-8 | ATHB-8 (HOMEOBOX GENE 8); DNA binding / transcription factor | chr4:15863456-15868629 REVERSE [PFAM] |
| submitted-miRNA | AT5G60690.1 | | Symbols: IFL, IFL1, REV | REV (REVOLUTA); DNA binding / lipid binding / transcription factor | chr5:24414249-24419421 FORWARD [PFAM] |
| submitted-miRNA | AT1G41820.1 | | Symbols: | unknown protein | chr1:15584864-15587584 REVERSE [PFAM] |
| submitted-miRNA | AT2G46020.1 | | Symbols: ATBRM, CHR2, BRM | ATBRM/CHR2 (ARABIDOPSIS THALIANA BRAHMA); ATP binding / DNA binding / helicase/ transcription regulator | chr2:18930377-18938842 FORWARD [PFAM] |
| submitted-miRNA | AT2G46020.2 | | Symbols: ATBRM, CHR2, BRM | ATBRM/CHR2 (ARABIDOPSIS THALIANA BRAHMA); ATP binding / DNA binding / helicase/ transcription regulator | chr2:18930377-18939007 FORWARD [PFAM] |
| submitted-miRNA | AT4G03250.1 | | Symbols: | homeobox-leucine zipper family protein | chr4:1425622-1427744 FORWARD [PFAM] |
| **8 potential target genes of miR166c** | | |
| sRNA_Acc. | Target_Acc. | Target_Desc. |
| submitted-miRNA | AT1G52150.1 | | Symbols: ATHB15, CNA, ICU4, ATHB-15 | ATHB-15 (INCURVATA 4); DNA binding / transcription factor | chr1:19413380-19418347 REVERSE [PFAM] 2782-3225 PF08670.4 MEKHLA domain; |
| submitted-miRNA | AT1G52150.2 | | Symbols: ATHB15, CNA, ICU4, ATHB-15 | ATHB-15 (INCURVATA 4); DNA binding / transcription factor | chr1:19413380-19418347 REVERSE [PFAM] 2785-3228 PF08670.4 MEKHLA domain; |
| submitted-miRNA | AT1G52150.3 | | Symbols: ATHB15, CNA, ICU4, ATHB-15 | ATHB-15 (INCURVATA 4) | chr1:19413380-19418347 REVERSE [PFAM] 2782-3081 PF08670.4 MEKHLA domain; |
| submitted-miRNA | AT1G30490.1 | | Symbols: ATHB9 | PHV (PHAVOLUTA); DNA binding / transcription factor | chr1:10796117-10800943 REVERSE [PFAM] |
| submitted-miRNA | AT2G34710.1 | | Symbols: ATHB14, ATHB-14, PHB-1D, PHB | PHB (PHABULOSA); DNA binding / transcription factor | chr2:14646402-14651352 REVERSE [PFAM] |
| submitted-miRNA | AT4G32880.1 | | Symbols: ATHB8, ATHB-8 | ATHB-8 (HOMEOBOX GENE 8); DNA binding / transcription factor | chr4:15863456-15868629 REVERSE [PFAM] |
| submitted-miRNA | AT5G60690.1 | | Symbols: IFL, IFL1, REV | REV (REVOLUTA); DNA binding / lipid binding / transcription factor | chr5:24414249-24419421 FORWARD [PFAM] |
| submitted-miRNA | AT5G49250.1 | | Symbols: | similar to unknown protein [Arabidopsis thaliana] (TAIR:AT4G29200.1); contains domain BETA-GALACTOSIDASE RELATED (PTHR23421); contains domain BETA-GALACTOSIDASE (PTHR23421:SF2) | chr5:19982569-19983171 FORWARD [PFAM] |
| **4 potential target genes of miR167a** | | |
| sRNA_Acc. | Target_Acc. | Target_Desc. |
| submitted-miRNA | AT3G04765.1 | | Symbols: MIR167C | MIR167C; miRNA | chr3:1306628-1306787 REVERSE [PFAM] |
| submitted-miRNA | AT3G22886.1 | | Symbols: MIR167A | MIR167A; miRNA | chr3:8108028-8108629 FORWARD [PFAM] |
| submitted-miRNA | AT3G61310.1 | | Symbols: | DNA-binding family protein | chr3:22701571-22703797 REVERSE [PFAM] |
| submitted-miRNA | AT5G41300.1 | | Symbols: | receptor-like protein kinase-related | chr5:16532232-16533330 FORWARD [PFAM] |
| **6 potential target genes of miR167b** | | |
| sRNA_Acc. | Target_Acc. | Target_Desc. |
| submitted-miRNA | AT3G04765.1 | | Symbols: MIR167C | MIR167C; miRNA | chr3:1306628-1306787 REVERSE [PFAM] |
| submitted-miRNA | AT3G22886.1 | | Symbols: MIR167A | MIR167A; miRNA | chr3:8108028-8108629 FORWARD [PFAM] |
| submitted-miRNA | AT5G37020.1 | | Symbols: ARF8 | ARF8 (AUXIN RESPONSE FACTOR 8); transcription factor | chr5:14647258-14651617 FORWARD [PFAM] 895-1128 PF06507.6 Auxin response factor; |
| submitted-miRNA | AT5G37020.2 | | Symbols: ARF8 | ARF8 (AUXIN RESPONSE FACTOR 8) | chr5:14647258-14651617 FORWARD [PFAM] 895-1128 PF06507.6 Auxin response factor; |
| submitted-miRNA | AT1G30330.1 | | Symbols: ARF6 | ARF6 (AUXIN RESPONSE FACTOR 6); transcription factor | chr1:10685804-10690781 REVERSE [PFAM] 1538-1771 PF06507.6 Auxin response factor; |
| submitted-miRNA | AT1G30330.2 | | Symbols: ARF6 | ARF6 (AUXIN RESPONSE FACTOR 6) | chr1:10685804-10690018 REVERSE [PFAM] 781-1014 PF06507.6 Auxin response factor; |
| **4 potential target genes of miR167c** | | |
| sRNA_Acc. | Target_Acc. | Target_Desc. |
| submitted-miRNA | AT3G04765.1 | | Symbols: MIR167C | MIR167C; miRNA | chr3:1306628-1306787 REVERSE [PFAM] |
| submitted-miRNA | AT3G22886.1 | | Symbols: MIR167A | MIR167A; miRNA | chr3:8108028-8108629 FORWARD [PFAM] |
| submitted-miRNA | AT3G61310.1 | | Symbols: | DNA-binding family protein | chr3:22701571-22703797 REVERSE [PFAM] |
| submitted-miRNA | AT5G41300.1 | | Symbols: | receptor-like protein kinase-related | chr5:16532232-16533330 FORWARD [PFAM] |
| **2 potential target genes of miR168a** | | |
| sRNA_Acc. | Target_Acc. | Target_Desc. |
| submitted-miRNA | AT1G48410.1 | | Symbols: AGO1 | AGO1 (ARGONAUTE 1) | chr1:17889766-17896254 REVERSE [PFAM] 1167-1316 PF08699.3 Domain of unknown function (DUF1785); |
| submitted-miRNA | AT1G48410.2 | | Symbols: AGO1 | AGO1 (ARGONAUTE 1) | chr1:17889766-17896254 REVERSE [PFAM] 1173-1322 PF08699.3 Domain of unknown function (DUF1785); |
| 1 potential target gene of miR168b | |  |
| sRNA_Acc. | Target_Acc. | Target_Desc. |
| submitted-miRNA | AT3G19740.1 | | Symbols: | ATPase | chr3:6855843-6859040 REVERSE [PFAM] |
| **6 potential target genes of miR171a** | | |
| sRNA_Acc. | Target_Acc. | Target_Desc. |
| submitted-miRNA | AT2G45160.1 | | Symbols: | scarecrow transcription factor family protein | chr2:18624937-18627225 REVERSE [PFAM] |
| submitted-miRNA | AT3G60630.1 | | Symbols: | scarecrow transcription factor family protein | chr3:22421340-22423583 REVERSE [PFAM] |
| submitted-miRNA | AT4G00150.1 | | Symbols: | scarecrow-like transcription factor 6 (SCL6) | chr4:57199-59286 REVERSE [PFAM] |
| submitted-miRNA | AT1G62035.1 | | Symbols: MIR171C | MIR171C; miRNA | chr1:22933754-22933869 REVERSE [PFAM] |
| submitted-miRNA | AT3G47170.1 | | Symbols: | transferase family protein | chr3:17379657-17381479 REVERSE [PFAM] |
| submitted-miRNA | AT5G23250.1 | | Symbols: | succinyl-CoA ligase (GDP-forming) alpha-chain, mitochondrial, putative / succinyl-CoA synthetase, alpha chain, putative / SCS-alpha, putative | chr5:7830363-7832846 FORWARD [PFAM] |
| **6 potential target genes of miR171b** | | |
| sRNA_Acc. | Target_Acc. | Target_Desc. |
| submitted-miRNA | AT2G45160.1 | | Symbols: | scarecrow transcription factor family protein | chr2:18624937-18627225 REVERSE [PFAM] |
| submitted-miRNA | AT3G60630.1 | | Symbols: | scarecrow transcription factor family protein | chr3:22421340-22423583 REVERSE [PFAM] |
| submitted-miRNA | AT4G00150.1 | | Symbols: | scarecrow-like transcription factor 6 (SCL6) | chr4:57199-59286 REVERSE [PFAM] |
| submitted-miRNA | AT1G62035.1 | | Symbols: MIR171C | MIR171C; miRNA | chr1:22933754-22933869 REVERSE [PFAM] |
| submitted-miRNA | AT3G47170.1 | | Symbols: | transferase family protein | chr3:17379657-17381479 REVERSE [PFAM] |
| submitted-miRNA | AT5G23250.1 | | Symbols: | succinyl-CoA ligase (GDP-forming) alpha-chain, mitochondrial, putative / succinyl-CoA synthetase, alpha chain, putative / SCS-alpha, putative | chr5:7830363-7832846 FORWARD [PFAM] |
| **15 potential target genes of miR395a** | | |
| sRNA_Acc. | Target_Acc. | Target_Desc. |
| submitted-miRNA | AT2G28780.1 | | Symbols: | similar to unknown protein [Arabidopsis thaliana] (TAIR:AT3G09450.1); similar to P-type trefoil [Medicago truncatula] (GB:ABD32899.1) | chr2:12347111-12350498 REVERSE [PFAM] |
| submitted-miRNA | AT5G10180.1 | | Symbols: SULTR2;1, AST68 | AST68 (Sulfate transporter 2.1) | chr5:3193145-3197121 FORWARD [PFAM] |
| submitted-miRNA | AT5G43780.1 | | Symbols: APS4 | APS4 | chr5:17606621-17608762 REVERSE [PFAM] |
| submitted-miRNA | AT1G50930.1 | | Symbols: | similar to unknown protein [Arabidopsis thaliana] (TAIR:AT3G20557.1); similar to conserved hypothetical protein [Medicago truncatula] (GB:ABE91163.1); contains domain UNCHARACTERIZED (PTHR23140:SF6); contains domain UNCHARACTERIZED (PTHR23140) | chr1:18880505-18881512 FORWARD [PFAM] |
| submitted-miRNA | AT5G13630.1 | | Symbols: CCH, CHLH, CCH1, GUN5 | GUN5 (GENOMES UNCOUPLED 5) | chr5:4387337-4392230 REVERSE [PFAM] |
| submitted-miRNA | AT5G13630.2 | | Symbols: CCH, CHLH, CCH1, GUN5 | GUN5 (GENOMES UNCOUPLED 5) | chr5:4387337-4392230 REVERSE [PFAM] |
| submitted-miRNA | AT1G69792.1 | | Symbols: MIR395D | MIR395D; miRNA | chr1:26273642-26273741 REVERSE [PFAM] |
| submitted-miRNA | AT1G69797.1 | | Symbols: MIR395F | MIR395F; miRNA | chr1:26277521-26277632 FORWARD [PFAM] |
| submitted-miRNA | AT4G23990.1 | | Symbols: CSLG3, ATCSLG3 | ATCSLG3 (Cellulose synthase-like G3); transferase/ transferase, transferring glycosyl groups | chr4:12456501-12460763 FORWARD [PFAM] |
| submitted-miRNA | AT1G26973.1 | | Symbols: MIR395A | MIR395A; miRNA | chr1:9363183-9363275 REVERSE [PFAM] |
| submitted-miRNA | AT1G69795.1 | | Symbols: MIR395E | MIR395E; miRNA | chr1:26276439-26276533 REVERSE [PFAM] |
| submitted-miRNA | AT2G17787.1 | | Symbols: | similar to unknown protein [Arabidopsis thaliana] (TAIR:AT4G35940.1); similar to conserved hypothetical protein [Medicago truncatula] (GB:ABE79676.1) | chr2:7738157-7740101 FORWARD [PFAM] |
| submitted-miRNA | AT2G20463.1 | | Symbols: | Encodes a defensin-like (DEFL) family protein. | chr2:8830814-8831203 REVERSE [PFAM] |
| submitted-miRNA | AT3G22890.1 | | Symbols: APS1 | APS1 (ATP sulfurylase 3) | chr3:8112730-8114997 FORWARD [PFAM] |
| submitted-miRNA | AT4G14680.1 | | Symbols: APS3 | APS3 (ATP sulfurylase 2); sulfate adenylyltransferase (ATP) | chr4:8413283-8415386 REVERSE [PFAM] |
| **17 potential target genes of miR395b** | | |
| sRNA_Acc. | Target_Acc. | Target_Desc. |
| submitted-miRNA | AT5G43780.1 | | Symbols: APS4 | APS4 | chr5:17606621-17608762 REVERSE [PFAM] |
| submitted-miRNA | AT1G50930.1 | | Symbols: | similar to unknown protein [Arabidopsis thaliana] (TAIR:AT3G20557.1); similar to conserved hypothetical protein [Medicago truncatula] (GB:ABE91163.1); contains domain UNCHARACTERIZED (PTHR23140:SF6); contains domain UNCHARACTERIZED (PTHR23140) | chr1:18880505-18881512 FORWARD [PFAM] |
| submitted-miRNA | AT1G69792.1 | | Symbols: MIR395D | MIR395D; miRNA | chr1:26273642-26273741 REVERSE [PFAM] |
| submitted-miRNA | AT1G69797.1 | | Symbols: MIR395F | MIR395F; miRNA | chr1:26277521-26277632 FORWARD [PFAM] |
| submitted-miRNA | AT2G28780.1 | | Symbols: | similar to unknown protein [Arabidopsis thaliana] (TAIR:AT3G09450.1); similar to P-type trefoil [Medicago truncatula] (GB:ABD32899.1) | chr2:12347111-12350498 REVERSE [PFAM] |
| submitted-miRNA | AT5G10180.1 | | Symbols: SULTR2;1, AST68 | AST68 (Sulfate transporter 2.1) | chr5:3193145-3197121 FORWARD [PFAM] |
| submitted-miRNA | AT1G26973.1 | | Symbols: MIR395A | MIR395A; miRNA | chr1:9363183-9363275 REVERSE [PFAM] |
| submitted-miRNA | AT1G69795.1 | | Symbols: MIR395E | MIR395E; miRNA | chr1:26276439-26276533 REVERSE [PFAM] |
| submitted-miRNA | AT3G22890.1 | | Symbols: APS1 | APS1 (ATP sulfurylase 3) | chr3:8112730-8114997 FORWARD [PFAM] |
| submitted-miRNA | AT4G14680.1 | | Symbols: APS3 | APS3 (ATP sulfurylase 2); sulfate adenylyltransferase (ATP) | chr4:8413283-8415386 REVERSE [PFAM] |
| submitted-miRNA | AT5G13630.1 | | Symbols: CCH, CHLH, CCH1, GUN5 | GUN5 (GENOMES UNCOUPLED 5) | chr5:4387337-4392230 REVERSE [PFAM] |
| submitted-miRNA | AT5G13630.2 | | Symbols: CCH, CHLH, CCH1, GUN5 | GUN5 (GENOMES UNCOUPLED 5) | chr5:4387337-4392230 REVERSE [PFAM] |
| submitted-miRNA | AT1G26975.1 | | Symbols: MIR395B | MIR395B; miRNA | chr1:9364458-9364557 FORWARD [PFAM] |
| submitted-miRNA | AT1G26985.1 | | Symbols: MIR395C | MIR395C; miRNA | chr1:9367067-9367166 FORWARD [PFAM] |
| submitted-miRNA | AT2G19540.1 | | Symbols: | transducin family protein / WD-40 repeat family protein | chr2:8468839-8471616 FORWARD [PFAM] |
| submitted-miRNA | AT4G23990.1 | | Symbols: CSLG3, ATCSLG3 | ATCSLG3 (Cellulose synthase-like G3); transferase/ transferase, transferring glycosyl groups | chr4:12456501-12460763 FORWARD [PFAM] |
| submitted-miRNA | AT3G13070.1 | | Symbols: | CBS domain-containing protein / transporter associated domain-containing protein | chr3:4191358-4195119 REVERSE [PFAM] |
| **5 potential target genes of miR396a** | | |
| sRNA_Acc. | Target_Acc. | Target_Desc. |
| submitted-miRNA | AT2G46060.1 | | Symbols: | transmembrane protein-related | chr2:18948172-18952172 REVERSE [PFAM] |
| submitted-miRNA | AT2G46060.2 | | Symbols: | transmembrane protein-related | chr2:18948172-18952129 REVERSE [PFAM] |
| submitted-miRNA | AT3G54280.1 | | Symbols: | ATP binding / DNA binding / helicase | chr3:20103339-20115133 FORWARD [PFAM] |
| submitted-miRNA | AT5G35407.1 | | Symbols: MIR396B | MIR396B; miRNA | chr5:13629028-13629162 FORWARD [PFAM] |
| submitted-miRNA | AT2G10606.1 | | Symbols: MIR396A | MIR396A; miRNA | chr2:4149405-4149555 REVERSE [PFAM] |
| **18 potential target genes of miR396b** | | |
| sRNA_Acc. | Target_Acc. | Target_Desc. |
| submitted-miRNA | AT5G35407.1 | | Symbols: MIR396B | MIR396B; miRNA | chr5:13629028-13629162 FORWARD [PFAM] |
| submitted-miRNA | AT2G10606.1 | | Symbols: MIR396A | MIR396A; miRNA | chr2:4149405-4149555 REVERSE [PFAM] |
| submitted-miRNA | AT5G01370.1 | | Symbols: | similar to unknown protein [Arabidopsis thaliana] (TAIR:AT2G22795.1); similar to hypothetical protein DDBDRAFT_0192009 [Dictyostelium discoideum AX4] (GB:XP_629009.1) | chr5:152529-154387 FORWARD [PFAM] |
| submitted-miRNA | AT5G43060.1 | | Symbols: | cysteine proteinase, putative / thiol protease, putative | chr5:17286735-17289392 REVERSE [PFAM] 459-1100 PF00112.16 Papain family cysteine protease; 195-377 PF08246.5 Cathepsin propeptide inhibitor domain (I29); |
| submitted-miRNA | AT2G34530.1 | | Symbols: | similar to binding [Arabidopsis thaliana] (TAIR:AT2G34540.2); contains InterPro domain Protein prenyltransferase; (InterPro:IPR008940) | chr2:14556057-14557324 REVERSE [PFAM] |
| submitted-miRNA | AT2G34530.2 | | Symbols: | unknown protein | chr2:14556168-14557320 REVERSE [PFAM] |
| submitted-miRNA | AT3G19400.1 | | Symbols: | cysteine proteinase, putative | chr3:6725474-6727006 FORWARD [PFAM] |
| submitted-miRNA | AT3G19400.2 | | Symbols: | cysteine proteinase, putative | chr3:6725474-6726584 FORWARD [PFAM] 430-882 PF00112.16 Papain family cysteine protease; |
| submitted-miRNA | AT5G57590.1 | | Symbols: BIO1 | BIO1 (BIOTIN AUXOTROPH 1) | chr5:23335734-23338765 REVERSE [PFAM] |
| submitted-miRNA | AT1G46696.1 | | Symbols: | similar to myosin heavy chain-related [Arabidopsis thaliana] (TAIR:AT4G03830.1); similar to hypothetical protein 26.t00052 [Brassica oleracea] (GB:ABD65035.1); contains InterPro domain Protein of unknown function DUF601; (InterPro:IPR006736) | chr1:17264300-17266623 REVERSE [PFAM] |
| submitted-miRNA | AT1G80060.1 | | Symbols: | similar to unknown protein [Arabidopsis thaliana] (TAIR:AT4G32270.1); similar to Ubiquitin [Medicago truncatula] (GB:ABE81772.1); similar to Os02g0188500 [Oryza sativa (japonica cultivar-group)] (GB:NP_001046135.1); contains domain no description (G3D.3.10.20.90); contains domain Ubiquitin-like (SSF54236) | chr1:30121102-30122481 REVERSE [PFAM] |
| submitted-miRNA | AT2G15630.1 | | Symbols: | pentatricopeptide (PPR) repeat-containing protein | chr2:6821603-6823486 FORWARD [PFAM] |
| submitted-miRNA | AT3G14110.1 | | Symbols: FLU | FLU (FLUORESCENT IN BLUE LIGHT); binding | chr3:4676125-4677665 REVERSE [PFAM] |
| submitted-miRNA | AT3G14110.2 | | Symbols: FLU | FLU (FLUORESCENT IN BLUE LIGHT); binding | chr3:4676125-4677665 REVERSE [PFAM] |
| submitted-miRNA | AT3G23190.1 | | Symbols: | lesion inducing protein-related | chr3:8279296-8280919 FORWARD [PFAM] |
| submitted-miRNA | AT4G12050.1 | | Symbols: | DNA-binding protein-related | chr4:7219818-7221322 REVERSE [PFAM] |
| submitted-miRNA | AT5G53220.1 | | Symbols: | similar to unknown protein [Arabidopsis thaliana] (TAIR:AT2G22795.1); similar to OSJNBb0076A22.19 [Oryza sativa (japonica cultivar-group)] (GB:CAD40807.1); similar to unknown protein [Oryza sativa (japonica cultivar-group)] (GB:BAD72405.1); similar to Os01g0221300 [Oryza sativa (japonica cultivar-group)] (GB:NP_001042431.1) | chr5:21613221-21615450 FORWARD [PFAM] |
| submitted-miRNA | AT5G53220.2 | | Symbols: | similar to unknown protein [Arabidopsis thaliana] (TAIR:AT2G22795.1); similar to OSJNBb0076A22.19 [Oryza sativa (japonica cultivar-group)] (GB:CAD40807.1); similar to unknown protein [Oryza sativa (japonica cultivar-group)] (GB:BAD72405.1); similar to Os01g0221300 [Oryza sativa (japonica cultivar-group)] (GB:NP_001042431.1) | chr5:21613216-21615450 FORWARD [PFAM] |
| **13 potential target genes of miR396c** | | |
| sRNA_Acc. | Target_Acc. | Target_Desc. |
| submitted-miRNA | AT5G35407.1 | | Symbols: MIR396B | MIR396B; miRNA | chr5:13629028-13629162 FORWARD [PFAM] |
| submitted-miRNA | AT2G10606.1 | | Symbols: MIR396A | MIR396A; miRNA | chr2:4149405-4149555 REVERSE [PFAM] |
| submitted-miRNA | AT2G34530.1 | | Symbols: | similar to binding [Arabidopsis thaliana] (TAIR:AT2G34540.2); contains InterPro domain Protein prenyltransferase; (InterPro:IPR008940) | chr2:14556057-14557324 REVERSE [PFAM] |
| submitted-miRNA | AT2G34530.2 | | Symbols: | unknown protein | chr2:14556168-14557320 REVERSE [PFAM] |
| submitted-miRNA | AT5G01370.1 | | Symbols: | similar to unknown protein [Arabidopsis thaliana] (TAIR:AT2G22795.1); similar to hypothetical protein DDBDRAFT_0192009 [Dictyostelium discoideum AX4] (GB:XP_629009.1) | chr5:152529-154387 FORWARD [PFAM] |
| submitted-miRNA | AT5G43060.1 | | Symbols: | cysteine proteinase, putative / thiol protease, putative | chr5:17286735-17289392 REVERSE [PFAM] 459-1100 PF00112.16 Papain family cysteine protease; 195-377 PF08246.5 Cathepsin propeptide inhibitor domain (I29); |
| submitted-miRNA | AT5G57590.1 | | Symbols: BIO1 | BIO1 (BIOTIN AUXOTROPH 1) | chr5:23335734-23338765 REVERSE [PFAM] |
| submitted-miRNA | AT1G46696.1 | | Symbols: | similar to myosin heavy chain-related [Arabidopsis thaliana] (TAIR:AT4G03830.1); similar to hypothetical protein 26.t00052 [Brassica oleracea] (GB:ABD65035.1); contains InterPro domain Protein of unknown function DUF601; (InterPro:IPR006736) | chr1:17264300-17266623 REVERSE [PFAM] |
| submitted-miRNA | AT2G15630.1 | | Symbols: | pentatricopeptide (PPR) repeat-containing protein | chr2:6821603-6823486 FORWARD [PFAM] |
| submitted-miRNA | AT4G12050.1 | | Symbols: | DNA-binding protein-related | chr4:7219818-7221322 REVERSE [PFAM] |
| submitted-miRNA | AT5G53220.1 | | Symbols: | similar to unknown protein [Arabidopsis thaliana] (TAIR:AT2G22795.1); similar to OSJNBb0076A22.19 [Oryza sativa (japonica cultivar-group)] (GB:CAD40807.1); similar to unknown protein [Oryza sativa (japonica cultivar-group)] (GB:BAD72405.1); similar to Os01g0221300 [Oryza sativa (japonica cultivar-group)] (GB:NP_001042431.1) | chr5:21613221-21615450 FORWARD [PFAM] |
| submitted-miRNA | AT5G53220.2 | | Symbols: | similar to unknown protein [Arabidopsis thaliana] (TAIR:AT2G22795.1); similar to OSJNBb0076A22.19 [Oryza sativa (japonica cultivar-group)] (GB:CAD40807.1); similar to unknown protein [Oryza sativa (japonica cultivar-group)] (GB:BAD72405.1); similar to Os01g0221300 [Oryza sativa (japonica cultivar-group)] (GB:NP_001042431.1) | chr5:21613216-21615450 FORWARD [PFAM] |
| submitted-miRNA | AT5G58980.1 | | Symbols: | ceramidase family protein | chr5:23828675-23831621 REVERSE [PFAM] |
| **11 potential target genes of miR397a** | | |
| sRNA_Acc. | Target_Acc. | Target_Desc. |
| submitted-miRNA | AT2G29130.1 | | Symbols: LAC2 | LAC2 (laccase 2); copper ion binding / oxidoreductase | chr2:12531966-12534776 REVERSE [PFAM] 1414-1659 PF07731.7 Multicopper oxidase; |
| submitted-miRNA | AT2G38080.1 | | Symbols: IRX12, LAC4 | IRX12/LAC4 (laccase 4); copper ion binding / oxidoreductase | chr2:15941538-15944694 FORWARD [PFAM] |
| submitted-miRNA | AT5G60020.1 | | Symbols: LAC17 | LAC17 (laccase 17); copper ion binding / oxidoreductase | chr5:24185222-24187688 FORWARD [PFAM] 164-508 PF07732.8 Multicopper oxidase; |
| submitted-miRNA | AT1G66670.1 | | Symbols: NCLPP4, CLPP3 | CLPP3 (Clp protease proteolytic subunit 3); endopeptidase Clp | chr1:24867448-24869365 REVERSE [PFAM] 306-830 PF00574.16 Clp protease; |
| submitted-miRNA | AT3G06040.1 | | Symbols: | ribosomal protein L12 family protein | chr3:1824382-1825669 REVERSE [PFAM] |
| submitted-miRNA | AT3G06040.2 | | Symbols: | ribosomal protein L12 family protein | chr3:1824382-1825634 REVERSE [PFAM] |
| submitted-miRNA | AT3G06040.3 | | Symbols: | ribosomal protein L12 family protein | chr3:1824491-1825648 REVERSE [PFAM] |
| submitted-miRNA | AT3G06470.1 | | Symbols: | GNS1/SUR4 membrane family protein | chr3:1984174-1985265 FORWARD [PFAM] |
| submitted-miRNA | AT4G33230.1 | | Symbols: | pectinesterase family protein | chr4:16026595-16028758 REVERSE [PFAM] 886-1779 PF01095.12 Pectinesterase; |
| submitted-miRNA | AT5G11210.1 | | Symbols: GLR2.5, ATGLR2.5 | ATGLR2.5 (Arabidopsis thaliana glutamate receptor 2.5) | chr5:3571215-3574538 REVERSE [PFAM] |
| submitted-miRNA | AT5G64020.1 | | Symbols: | similar to unknown protein [Arabidopsis thaliana] (TAIR:AT2G37720.1); similar to leaf senescence protein-like [Oryza sativa (japonica cultivar-group)] (GB:BAD28782.1); similar to Os09g0397400 [Oryza sativa (japonica cultivar-group)] (GB:NP_001063105.1); contains InterPro domain Protein of unknown function DUF231, plant; (InterPro:IPR004253) | chr5:25637534-25639799 REVERSE [PFAM] |
| **5 potential target genes of miR398a** | | |
| sRNA_Acc. | Target_Acc. | Target_Desc. |
| submitted-miRNA | AT5G14550.1 | | Symbols: | similar to unknown protein [Arabidopsis thaliana] (TAIR:AT1G62305.1); similar to Os01g0695200 [Oryza sativa (japonica cultivar-group)] (GB:NP_001043958.1); similar to Protein of unknown function DUF266, plant [Medicago truncatula] (GB:ABD28621.1); similar to Os05g0170000 [Oryza sativa (japonica cultivar-group)] (GB:NP_001054766.1); contains InterPro domain Protein of unknown function DUF266, plant; (InterPro:IPR004949) | chr5:4691013-4694058 REVERSE [PFAM] |
| submitted-miRNA | AT5G14545.1 | | Symbols: MIR398B | MIR398B; miRNA | chr5:4691025-4691140 FORWARD [PFAM] |
| submitted-miRNA | AT5G14565.1 | | Symbols: MIR398C | MIR398C; miRNA | chr5:4694629-4697016 FORWARD [PFAM] |
| submitted-miRNA | AT1G36078.1 | | Symbols: | unknown protein | chr1:13482877-13483997 REVERSE [PFAM] |
| submitted-miRNA | AT3G06370.1 | | Symbols: ATNHX4, NHX4 | NHX4 (sodium proton exchanger 4); sodium:hydrogen antiporter | chr3:1930402-1934079 REVERSE [PFAM] |
| **3 potential target genes of miR398b** | | |
| sRNA_Acc. | Target_Acc. | Target_Desc. |
| submitted-miRNA | AT5G14550.1 | | Symbols: | similar to unknown protein [Arabidopsis thaliana] (TAIR:AT1G62305.1); similar to Os01g0695200 [Oryza sativa (japonica cultivar-group)] (GB:NP_001043958.1); similar to Protein of unknown function DUF266, plant [Medicago truncatula] (GB:ABD28621.1); similar to Os05g0170000 [Oryza sativa (japonica cultivar-group)] (GB:NP_001054766.1); contains InterPro domain Protein of unknown function DUF266, plant; (InterPro:IPR004949) | chr5:4691013-4694058 REVERSE [PFAM] |
| submitted-miRNA | AT5G14545.1 | | Symbols: MIR398B | MIR398B; miRNA | chr5:4691025-4691140 FORWARD [PFAM] |
| submitted-miRNA | AT5G14565.1 | | Symbols: MIR398C | MIR398C; miRNA | chr5:4694629-4697016 FORWARD [PFAM] |
