# Supplementary material for: Differential miRNA expression in Rehmannia glutinosa plants subjected to continuous cropping
Source: BMC Plant Biol. 2011 Mar 26;11:53. doi: 10.1186/1471-2229-11-53 (PMC3078876; doi:10.1186/1471-2229-11-53)
Supplement: Additional file 4 — Sequence alignments of partial targets of differential expressed miRNAs. [file 1471-2229-11-53-S4.DOC]

**Additional file 4 - Sequence alignments of partial targets of differential expressed miRNAs.**

miR160 target - ARF16

miR167 target - ARF6

miR5138 target - ICU2

miR5140 targett - a gene of magnesium transporter CorA-like family protein
